# Simultaneously Achieving Efficient Narrow‐Band Emission and Large Emission Dissymmetry Factor in an Achiral Hybrid Indium Chloride

**DOI:** 10.1002/advs.202522728

**Published:** 2026-01-22

**Authors:** Haowei Lin, Abdusalam Ablez, Xinping Guo, Yingchen Peng, Jiance Jin, Zhihua Chen, Kezhao Du, Zeping Wang, Xiaoying Huang

**Affiliations:** ^1^ State Key Laboratory of Structural Chemistry Fujian Institute of Research on the Structure of Matter The Chinese Academy of Sciences Fuzhou Fujian P. R. China; ^2^ University of Chinese Academy of Sciences Beijing P. R. China; ^3^ Fujian College University of Chinese Academy of Sciences Fuzhou Fujian P. R. China; ^4^ College of Chemistry Fuzhou University Fuzhou Fujian P. R. China; ^5^ College of Chemistry and Materials Science Fujian Provincial Key Laboratory of Advanced Materials Oriented Chemical Engineering Fujian Normal University Fuzhou P. R. China

**Keywords:** achiral space group, circularly polarized luminescence, indium halide, narrow‐band emission

## Abstract

To achieve high contrast in three‐dimensional display, circularly polarized luminescence (CPL) materials should simultaneously have narrow full width at half‐maximum (FWHM), high photoluminescence quantum yield (PLQY), and large dissymmetry factor (*g*_lum_). However, very few materials can fulfill it. Herein, we report a zero‐dimensional hybrid indium chloride [BPy][InCl_4_(dtbp)] (BPy = *N*‐butylpyridinium, dtbp = 4,4’‐di‐tert‐butyl‐2,2’‐dipyridyl). Although crystallizing in the achiral point group *mm*2, the compound demonstrates remarkable chiroptical activity. It shows efficient blue‐green emission peaking at 481 nm with a FWHM of 49 nm. The coordination with In^3+^ in the inorganic unit, combined with abundant intermolecular interactions, effectively suppresses vibrational relaxation of the organic ligand dtbp, resulting in narrow‐band emission. Moreover, owing to the synergy between this structural rigidity and the distortion of the InCl_4_(dtbp) unit, this compound overcomes the trade‐off between PLQY and *g*_lum_, exhibiting a PLQY approaching 100% and a |*g*_lum_| value of 0.283. Photophysical characterizations and density‐functional theory calculations indicate that the high‐efficiency emission originates from the ligand. As a rare example of optically active achiral crystals, this compound not only offers a promising candidate for CPL materials integrating narrow FWHM, high PLQY, and large *g*_lum_, but also provides valuable insight into the chiroptical properties of achiral crystalline systems.

## Graphical abstract

This work demonstrates that efficient CPL, characterized by high PLQY, large *g_lum_*, and narrow‐band emission, can be achieved simultaneously in a ligand‐modified hybrid indium chloride crystallizing in an achiral space group.

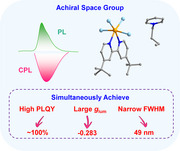

## 1 Introduction

Circularly polarized luminescence (CPL) materials, capable of directly emitting circularly polarized light without external optical elements such as polarizers, hold significant promise for applications in three‐dimensional (3D) imaging [1, 2], information encryption [3], and polarization‐based bio‐imaging [4, 5]. CPL material should simultaneously exhibit a large circular polarization luminescence dissymmetry factor (*g*_lum_) and a high photoluminescence quantum yield (PLQY), thus achieving a high signal‐to‐noise ratio [6, 7, 8, 9]. The former represents the polarization purity of the emitted light, while the latter indicates the efficiency of the energy conversion. However, due to the common mismatch between the electric dipole moment (*µ*) and the magnetic transition dipole moment (*m*), it is challenging to simultaneously achieve high PLQY and large *g*_lum_ in a CPL material [9, 10]. In addition, for achieving a high‐contrast display, realizing narrow‐band emission (i.e., small full width at half maximum (FWHM) in CPL materials is crucial [11, 12, 13, 14].

In recent years, zero‐dimensional (0‐D) organic‐inorganic hybrid metal halides (OIMHs) have emerged as luminescent materials with excellent performance in information storage, anti‐counterfeiting, and solid‐state lighting [15, 16, 17, 18, 19, 20]. 0‐D OIMHs with quantum confinement effects and strong electron‐phonon coupling usually display self‐trapped exciton emission (STEs) with high PLQY, large Stokes shifts, and large FWHM [21, 22]. While the high PLQY is advantageous for CPL applications, these materials face two intertwined challenges [23, 24, 25]. First, similar to other CPL materials, the mismatch between the electrical and magnetic transition dipoles hinders the simultaneous realization of a high PLQY and a large *g*_lum_ [10]. Second, and more critically for display technologies, the inherently broad FWHM of STE emission fundamentally limits color purity and contrast ratio, thus hindering their application in high‐performance 3D display. To overcome these limitations, it is imperative to develop novel OIMHs with rationally designed structures that can circumvent the broadband STE emission, for instance, by harnessing alternative luminescent centers capable of narrow‐band emission.

The incorporation of organic ligands into 0‐D OIMHs offers a powerful strategy to engineer their photophysical properties by forming ligand‐modified hybrid halide anions [22, 26, 27]. In such architectures, the organic ligands can function as efficient luminescent centers. Crucially, their integration into a rigid inorganic framework via coordination bonds effectively suppresses vibrational relaxation, which not only enhances the luminescence efficiency but also holds promise for achieving narrow‐band emission. Furthermore, the significant disparity in coordination geometry and ionic radii between halogens and the multidentate organic ligands induces substantial distortion in the metal‐halide octahedra [28]. This structural distortion is anticipated to break the spatial symmetry of the emissive centers, thereby providing a potential pathway to enhance the *g*_lum_ [29, 30].

Herein, we present a 0‐D In(III)‐based OIMH, namely [BPy][InCl_4_(dtbp)] (BPy = *N*‐butylpyridinium, dtbp = 4,4’‐di‐tert‐butyl‐2,2’‐dipyridyl), engineered through the organic components synergy strategy. It exhibits blue‐green luminescence with an emission peak located at 481 nm under 367 nm excitation. Impressively, it achieves a narrow FWHM of only 49 nm alongside a PLQY of close to 100%, a combination that is rare among OIMHs. Detailed photophysical characterization and density‐functional theory (DFT) calculations indicate that the emission of the compounds originates from the ligand dtbp. Structural analysis reveals that the dtbp ligands are rigidified by two key factors: their coordination to the inorganic [InCl_4_]^−^ moiety and extensive intermolecular interactions within the crystal lattice. This rigid environment effectively suppresses vibrational relaxation in the excited state, rationalizing the observed high‐efficiency narrow‐band emission. Remarkably, although the compound crystallizes in the achiral orthorhombic space group *Pca*2_1_ (point group *mm*2), it exhibits significant CPL with a large |*g*_lum_| of 0.283. The unique crystal structure of [BPy][InCl_4_(dtbp)], which simultaneously imposes structural rigidity and breaks emission symmetry, is therefore directly responsible for its excellent CPL performance. This work provides a novel design paradigm for achieving high‐performance CPL materials that concurrently possess narrow FWHM, high PLQY, and large *g*_lum_ in a single system.

## 2 Results and Discussion

**FIGURE 1:**
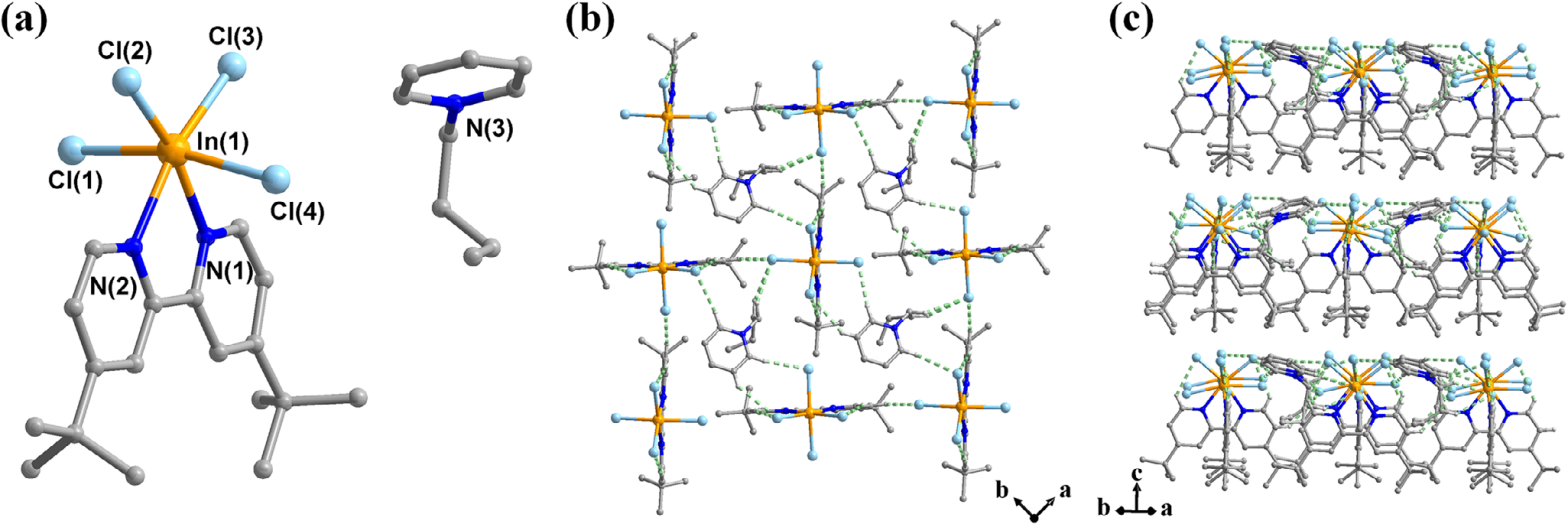
Structural diagrams for compound [BPy][InCl_4_(dtbp)]. (a) The asymmetric unit. (b) A two‐dimensional supramolecular layer formed by hydrogen bonding (green dashed lines) extending along the *ab* plane as viewed along the *c*‐axis. (c) Packing of two‐dimensional supramolecular layers along the *c*‐axis. In all figures, gray atoms represent carbon atoms and hydrogen atoms (except those forming H‐bonds in (b) and (c)), and one set of disordered carbon atoms is omitted for clarity.

The crystals of [BPy][InCl_4_(dtbp)] were synthesized using the solvothermal method (details are given in the Experimental Section). Single crystal X‐ray diffraction (SCXRD) was performed at room temperature (293 K), and the crystallographic data are listed in Table S1. [BPy][InCl_4_(dtbp)] crystallizes in the orthorhombic space group *Pca*2_1_ with cell parameters of *a* = 12.72960(10), *b* = 11.8279(2), *c* = 20.5592(3) Å, *V* = 3095.48(7) Å^3^, and *Z* = 4 (Table S1). The asymmetric unit comprises one [BPy]^+^ cation and one [InCl_4_(dtbp)]^−^ anion (Figure 1a and Figure S1). In the anion, the In(III) center adopts a distorted octahedral geometry, chelated by one dtbp ligand through its two nitrogen atoms and coordinated by four chloride ions. The In─Cl bond lengths range from 2.4431(12) to 2.5129(10) Å, comparable to those of previously reported compounds (Table S2) [31, 32]. The In–N bond lengths range from 2.310(4) to 2.307(4) Å, which are comparable to those of the previously reported compound [Ammim][InCl_4_(dmbp)] (Ammim = 1‐allyl‐2,3‐dimethylimidazolium, dmbp = 4,4’‐dimethyl‐2,2’‐bipyridyl) [31]. As shown in Figure 1b,c, the [BPy]^+^ cation is located between four [InCl_4_(dtbp)]^−^ anions, separating anions and balancing the charges to form a 0‐D structure. Interestingly, the structure is rich in C─H···Cl hydrogen bonding interactions between the [BPy]^+^ cation and the [InCl_4_(dtbp)]^−^ anion, forming a two‐dimensional supramolecular layered structure in the *ab* plane (Figure 1b). These layers then stack along the *c*‐axis, primarily through van der Waals interactions (Figure 1c and Figure S2). The hydrogen bonds and symmetry operations mentioned above are shown in Table S3.

The purity and stability of [BPy][InCl_4_(dtbp)] were confirmed by powder X‐ray diffraction (PXRD) and thermogravimetric (TG) analysis, respectively, as shown in Figures S3 and S4. The experimental PXRD pattern of [BPy][InCl_4_(dtbp)] powders is in agreement with the simulated one (Figure S3), confirming the purity and uniformity of the as‐synthesized sample. TG analysis shows that there is no weight loss below 230°C, indicating the good thermal stability of the title compound, which lays a foundation for the subsequent study of fluorescence properties (Figure S4).

**FIGURE 2:**
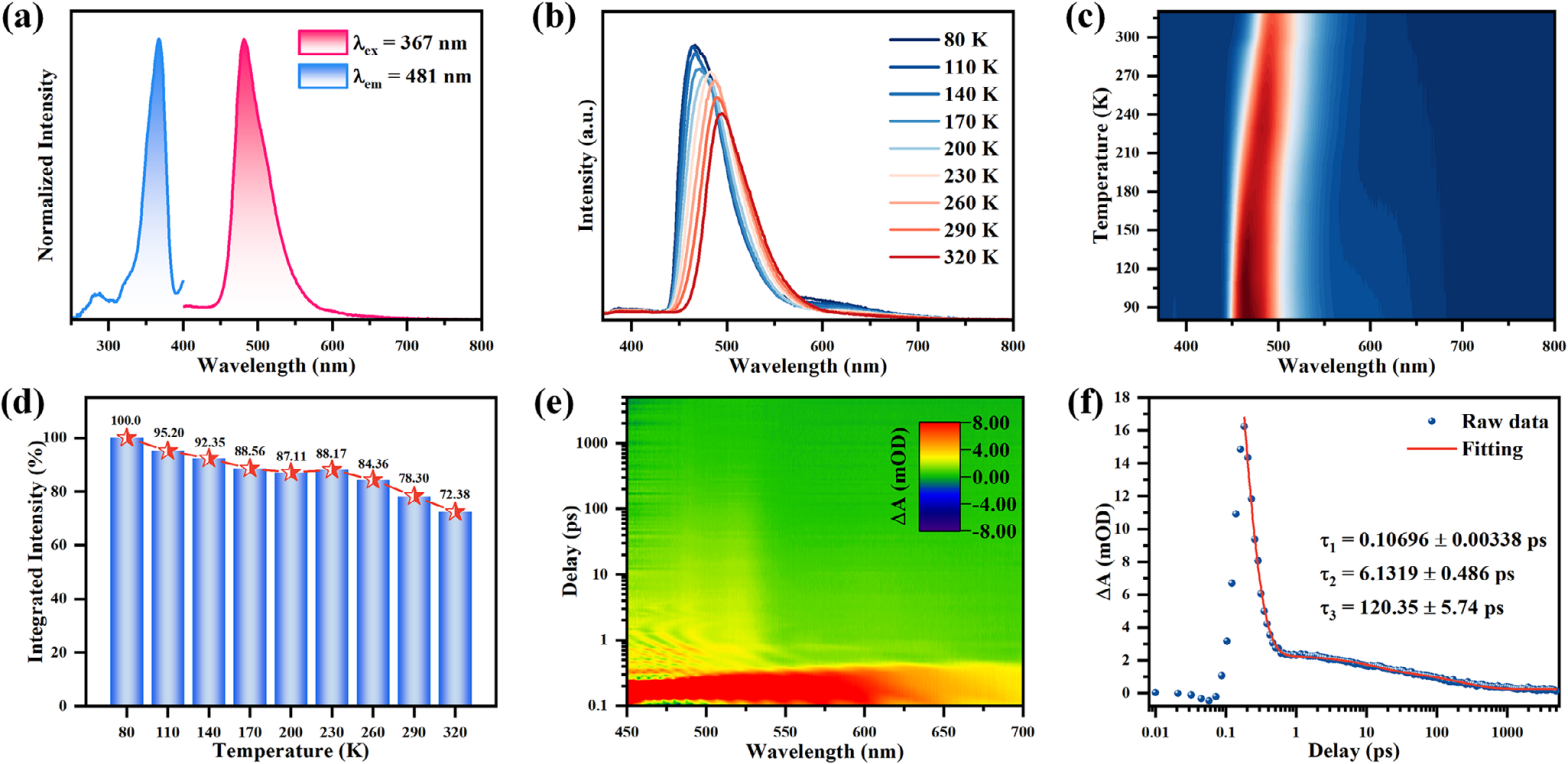
(a) PL excitation and emission spectra of [BPy][InCl_4_(dtbp)] at 300 K. (b) Temperature‐dependent PL spectra of [BPy][InCl_4_(dtbp)] under 367 nm excitation. (c) Temperature‐dependent PL spectra contour map of [BPy][InCl_4_(dtbp)] under 367 nm excitation. (d) Comparison of PL intensity at different temperatures of [BPy][InCl_4_(dtbp)]. (e) Pseudo‐color plot of fs‐TA spectrum of [BPy][InCl_4_(dtbp)] upon photoexcitation at 400 nm. (f) PIA decay and fitting curves of [BPy][InCl_4_(dtbp)] probed at 520 nm.

The photoluminescence (PL) properties of [BPy][InCl_4_(dtbp)] were investigated at room temperature. As shown in Figure 2a, [BPy][InCl_4_(dtbp)] emits light from 440 to 650 nm under 367 nm excitation at room temperature, producing a maximum peak at 481 nm (Figure 2a). Remarkably, at room temperature, it achieves PLQY of approximately 100% and possesses an emission lifetime of 4.878 ns (Figure S5). To gain further insight into the emission behavior, temperature‐dependent PL spectra were collected from 80 to 320 K (Figure 2b,c). The PL intensity increases progressively as the temperature decreases, which can be rationalized by the suppression of non‐radiative decay pathways at lower temperatures. Concurrently, the emission peak displays a notable blue shift from 497 nm at 320 K to 475 nm at 80 K. This blue shift is characteristic of suppressed electron‐phonon coupling at cryogenic temperatures [33]. Importantly, [BPy][InCl_4_(dtbp)] demonstrates exceptional antithermal quenching of its emission. The emission intensity of [BPy][InCl_4_(dtbp)] does not decrease significantly with increasing temperature, and it still maintains 80% of its strength at 80 to 320 K. This demonstrates that [BPy][InCl_4_(dtbp)] exhibits excellent resistance to thermal quenching (Figure 2d). This outstanding resistance to heat quenching can be attributed to the rigid structure of [BPy][InCl_4_(dtbp)].

To gain profound insights into the charge dynamics of [BPy][InCl_4_(dtbp)], femtosecond transient absorption (fs‐TA) spectroscopy was conducted for the single crystal sample. The sample was excited by a monochromatic pump pulse with a wavelength of 400 nm and probed by a super‐continuum white pulse in the visible region; the transmitted probe pulse was collected by a detector to measure the absorption change. As shown in Figure 2e, a compound in the ground state transforms to an excited state after absorbing excitation energy and generates an excited state absorption signal. As shown in Figure 2f, the PIA decay curve can be fitted by a three‐exponential function with time constants of τ_1_ = 0.10696 ps, τ_2_ = 6.1319 ps, and τ_3_ = 120.35 ps. The ultrafast component (τ_1_) can be attributed to the charge transfer process from inorganic In‐Cl to organic dtbp ligand. The middle component (τ_2_) corresponds to phonon‐mediated decay pathways. The long lifetime component (τ_3_) corresponds to the exciton recombination process from the dtbp ligand, along with the PL emission.

**FIGURE 3:**
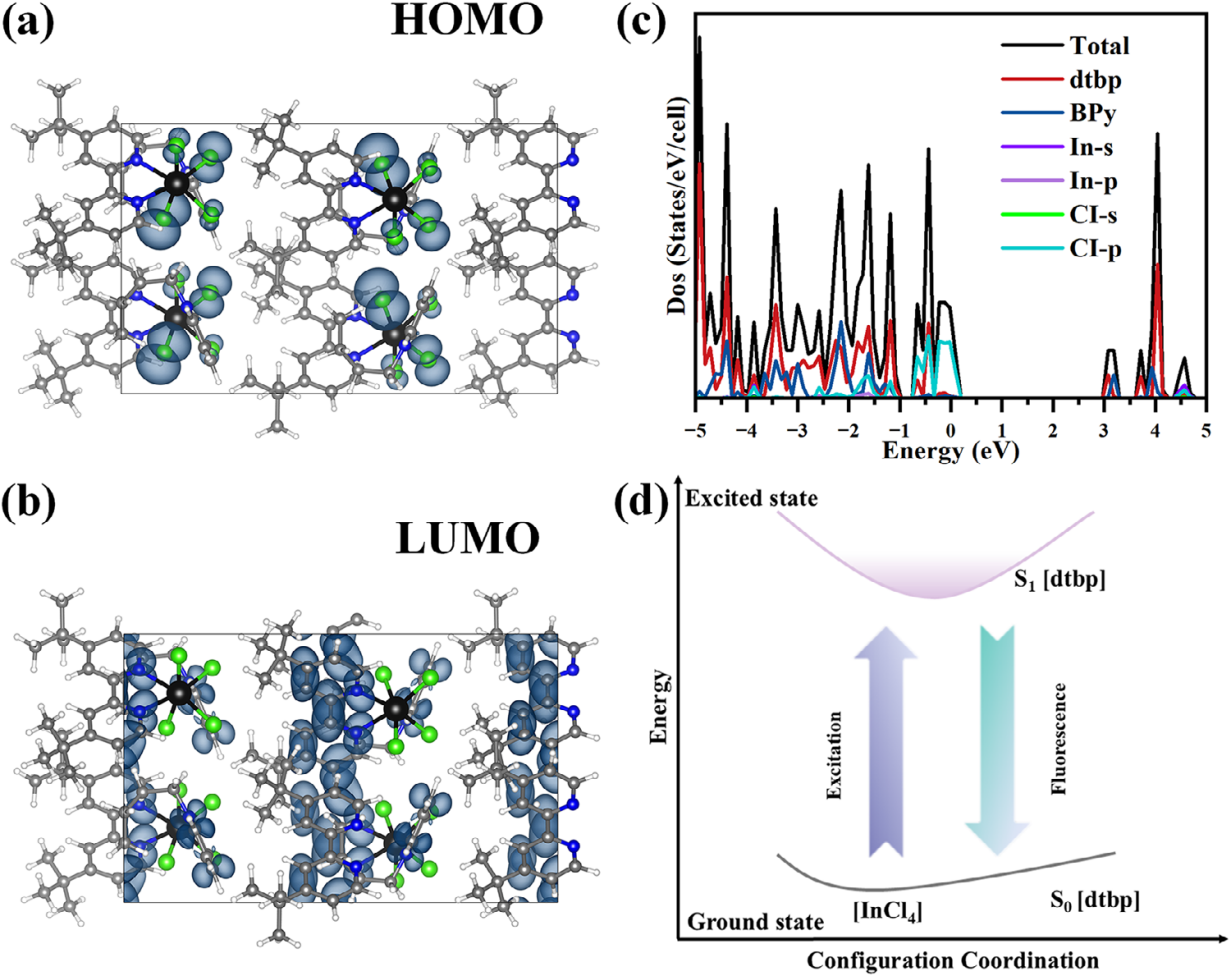
(a) Electron distribution map on the highest occupied molecular orbital (HOMO) of [BPy][InCl_4_(dtbp)]. (b) Electron distribution map on the lowest occupied molecular orbital (LUMO) of [BPy][InCl_4_(dtbp)]. (c) Density of states for [BPy][InCl_4_(dtbp)]. (d) Diagram of luminescent mechanism for [BPy][InCl_4_(dtbp)].

First‐principles calculations based on DFT were used to study the title compound's optical properties in depth. We calculated the highest occupied molecular orbital (HOMO) and lowest unoccupied molecular orbital (LUMO) of [BPy][InCl4(dtbp)] and the organic ligand dtbp (Figure 3a,b and Figure S6). Unlike the organic ligand, whose electron cloud is primarily concentrated on the six‐membered ring of bipyridine, the electron cloud of the title compound's HOMO is located on the Cl atoms, while the electron cloud of the LUMO is located on the dtbp ligand and [BPy]^+^ cation portions. The computational data demonstrate that [BPy][InCl_4_(dtbp)] is an indirect bandgap semiconductor with a bandgap value of 2.974 eV (Figure S7), which is slightly smaller than the bandgap value (3.534 eV, Figure S8) experimentally determined from the UV–vis absorption spectrum. This discrepancy arises from the insufficient precision of the GGA in characterising the eigenvalues of the electronic states [34, 35]. For [BPy][InCl_4_(dtbp)], the valence band maximum (VBM) is mainly contributed by Cl 3p, whereas the main composition of the conduction band minimum (CBM) is determined by the dtbp ligand and [BPy]^+^ cations (Figure 3c). These results indicate that the introduction of the inorganic halide unit results in halogen‐organic charge transfer in [BPy][InCl_4_(dtbp)].

The possible luminescence mechanism is as follows: under excitation, electrons transfer from the inorganic moiety (chloride ions) to the singlet excited state of the dtbp ligand, whereupon electrons in the singlet excited state of the dtbp ligand are emitted in the form of fluorescence (Figure 3d).

In stark contrast to the broad emission typically originating from STEs in most zero‐dimensional OIMHs, [BPy][InCl_4_(dtbp)] exhibits rare narrow‐band emission via its ligand‐based luminescent center. As summarized in Table S4, it represents the first main‐group zero‐dimensional metal halide to concurrently achieve a near‐unity PLQY and a narrow FWHM [31, 32, 36, 37, 38, 39, 40, 41, 42, 43, 44, 45, 46, 47]. To understand the origin of the narrow band emission of [BPy][InCl_4_(dtbp)], we tested the steady‐state luminescence spectra of the dtbp powders for comparison. Under the excitation at 350 nm, dtbp exhibits emission with a peak located at 535 nm and a FWHM of 107 nm (Figure S9). PLE of [BPy][InCl_4_(dtbp)] is highly consistent with that of dtbp, suggesting that the emission of [BPy][InCl_4_(dtbp)] may originate from the dtbp ligand. Time‐resolved PL spectroscopy shows that the luminescence lifetime decay of dtbp was about 1.413 ns (Figure S10), which is in good agreement with the decay value of 4.878 ns for [BPy][InCl_4_(dtbp)], which further supports the view that the luminescence of the title compound is mainly from the dtbp part. Additionally, compared to the ligand dtbp, [BPy][InCl_4_(dtbp)] exhibits a significant blue shift in the emission peak.

To investigate the structural origin of the narrow‐band emission and the observed blue shift in [BPy][InCl_4_(dtbp)], we compared the structures of [BPy][InCl_4_(dtbp)] with the dtbp ligand. The dtbp ligand crystallizes in the monoclinic crystal system with space group *P*2_1_/*c*, with unit cell parameters *a* = 10.241(5), *b* = 6.228(3), *c* = 24.559(10), *β* = 109.006(2)°, *V* = 1543.7(12) Å^3^, and *Z* = 4 (Table S5) [48]. In the structure of [BPy][InCl_4_(dtbp)], the dtbp ligand forms a coordination bond with the In^3+^ ion, which further enhances the rigidity of the ligand and effectively suppresses its vibrational relaxation. Furthermore, the incorporation of halide ions and organic cations into the structure introduces a significant number of C─H···Cl hydrogen bonds relative to the ligands themselves, thereby enhancing the intermolecular interactions of the title compounds compared to the ligands. To quantitatively assess this packing effect, we performed energy framework analysis. The results unambiguously demonstrate that [BPy][InCl_4_(dtbp)] possesses a more extensive and robust intermolecular interaction network, as visualized by the thicker cylindrical rods in its energy framework diagram (Figure S11). Furthermore, the calculated interaction energy (*E*_int_) for the title compound (−409.4 kcal mol^−^^1^) is more negative than that of the ligand dtbp (−137.8 kcal mol^−^^1^), consistent with enhanced intermolecular stabilization in [BPy][InCl_4_(dtbp)] (Table S6). The negative values signify energetically favorable binding processes, indicating spontaneity as the molecules assemble from infinity into the current configuration.

To sum up, the narrow‐band emission, blue‐shifted emission peaks, and enhanced PLQY of [BPy][InCl_4_(dtbp)] relative to the free dtbp ligand (FWHM: 49 nm vs. 107 nm; emission peak: 481 nm vs. 535 nm; PLQY: ∼100% vs. 12.96%) are attributed to a structural rigidity effect. This effect arises from two complementary structural factors: (1) the coordination of the dtbp ligand to the inorganic [InCl_4_]^−^ moiety, which imposes intrinsic geometric constraints, and (2) the extensive network of C─H···Cl hydrogen bonds involving the [BPy]^+^ cations, which creates a rigid supramolecular framework. Together, these factors drastically suppress the vibrational relaxation of the luminescent dtbp ligand in the excited state, thereby narrowing the emission bandwidth and boosting the radiative recombination efficiency.

**FIGURE 4:**
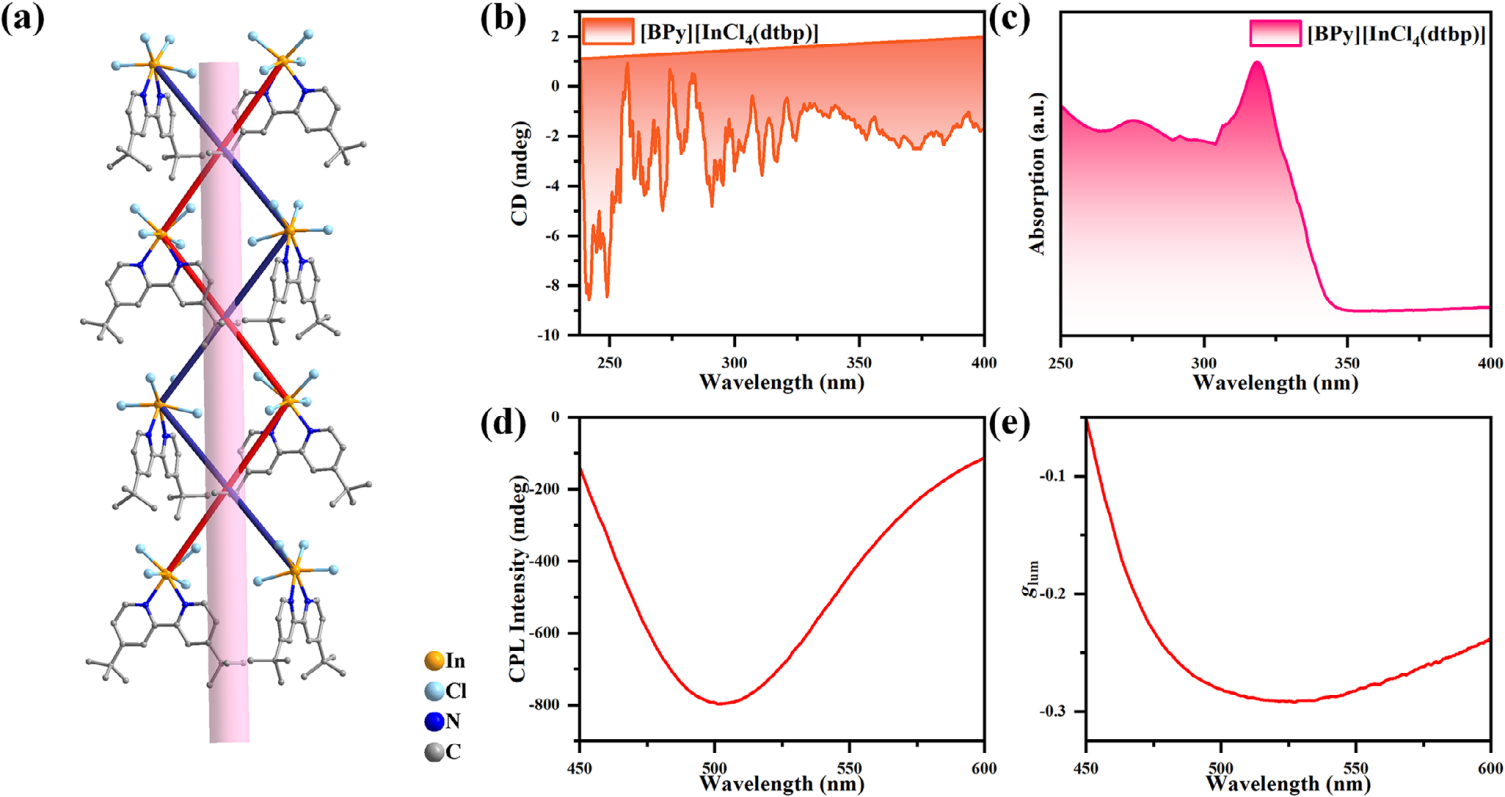
(a) Schematic diagram of the helical symmetry of [InCl_4_(dtbp)]^−^ anions on the 2_1_‐axis. Hydrogen atoms have been omitted for clarity. The pink columns represent the 2_1_‐axis, and the pale red and deep purple lines represent the direction of rotation. (b) CD spectra of [BPy][InCl_4_(dtbp)]. (c) UV absorption spectra of [BPy][InCl_4_(dtbp)]. (d) CPL spectra and (e) the corresponding *g*_lum_ of [BPy][InCl_4_(dtbp)].

Traditional views hold that chiroptical activity, such as CPL, is exclusive to chiral crystals. However, this is not entirely accurate. Beyond the 11 chiral point groups, four achiral point groups ($\bar{4}$2*m*, $\bar{4}$, *mm*2, and *m*) can also exhibit intrinsic chiroptical activity as dictated solely by their symmetry [49, 50, 51, 52]. Despite this theoretical possibility, crystalline materials belonging to these point groups are exceedingly rare, impeding detailed studies of their CPL properties [53]. This scarcity is particularly pronounced in the field of OIMHs, where structures with the non‐chiral *mm*2 point group constitute a mere 0.3% of reported compounds, and crucially, the realization of CPL in such *mm*2 systems has remained unreported until now (Table S7) [54, 55, 56, 57, 58, 59]. Our compound, [BPy][InCl_4_(dtbp)], which crystallizes in the orthorhombic space group *Pca*2_1_ (point group *mm*2), presents a rare opportunity to investigate this phenomenon. Structural analysis reveals that the [InCl_4_(dtbp)]^−^ anions are arranged in an intrinsically chiral double‐helical fashion around the 2_1_‐axis (Figure 4a). Motivated by this unique structure, we probed its chiroptical properties. Circular dichroism (CD) measurements revealed a distinct Cotton effect in the 250–400 nm range, which is consistent with the UV‐vis absorption spectrum (Figure 4b,c). This confirms the existence of ground‐state chirality. Subsequently, we investigated the excited‐state chirality using CPL spectroscopy.

**FIGURE 5:**
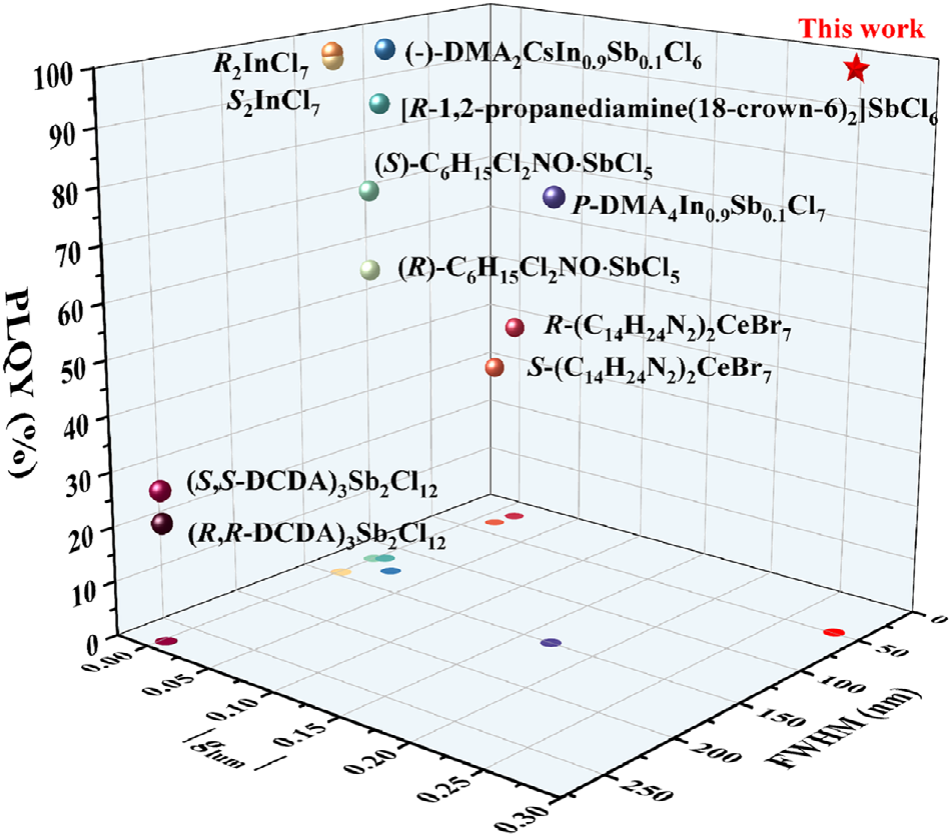
Comparison of PLQY, *g*_lum_, and FWHM of some reported 0‐D OIMHs with the present work. To facilitate comparison, the projection of the 3D image onto the *xy* plane is displayed on the *xy* plane.

Subsequently, we investigated the CPL performance of [BPy][InCl_4_(dtbp)] single crystals at room temperature. In general, *g*_lum_ can be used to visually assess the CPL performance of chiral luminescent materials. The following equation can calculate *g*_lum_: *g*_lum_ = 2 × (*I_L—_I_R_*) / (*I_L_* + *I_R_*), where *I_L_* and *I_R_* are the intensities of left‐ and right‐rotated circularly polarized light, respectively [9]. As shown in Figure 4d, the compounds exhibited CPL signals at 500 nm with a maximum *g*_lum_ value of ‐0.283 at 365 nm excitation wavelength (Figure 4e). The emission peaks of the CPL spectra were in perfect agreement with those of the fluorescence. Notably, the CPL performance of [BPy][InCl_4_(dtbp)] is exceptional when benchmarked against other reported 0‐D OIMHs. As summarized in Figure 5 and Table S8, this compound achieves a rare and optimal balance among a narrow FWHM (49 nm), a near‐unity PLQY (∼100%), and a large |*g*_lum_| value (0.283) [23, 24, 25, 60, 61, 62]. This integrated performance surpasses that of intrinsic narrow‐band emitters like hybrid rare‐earth halides and many 0‐D OIMHs constructed with chiral organic cations, positioning our material as a top‐tier candidate for CPL applications.

The exceptional balance between a near‐unity PLQY narrowband emission and a large *g*_lum_ value in [BPy][InCl_4_(dtbp)] is fundamentally rooted in its distinct crystal structure. The rigid microenvironment, enforced by extensive intermolecular interactions (e.g., C─H···Cl hydrogen bonds), plays a dual role. First, it severely suppresses non‐radiative decay pathways by restricting intramolecular vibrations and enhancing lattice stability, thereby yielding the high PLQY. Second, this structural rigidity stabilizes the unique geometric configuration of the excited‐state luminescent center and the vibrational relaxation of the excited‐state luminescent dtbp ligand, thereby narrowing the emission bandwidth. Furthermore, the significant disparity in coordination geometry and ionic radii between the chloride ions and the dtbp ligand induces substantial distortion in the [InCl_4_(dtbp)]^−^ octahedron. This structural distortion effectively lowers the local symmetry of the emissive center, which further promotes the pronounced chiral optical response. This compound provides a new idea for the future design and synthesis of OIMHs with efficient CPL properties.

## 3 Conclusions

In conclusion, we have successfully synthesized a 0‐D indium(III)‐based hybrid halide, [BPy][InCl_4_(dtbp)], via a rational organic components synergy strategy. This compound exhibits outstanding integrated optoelectronic properties, including brilliant blue‐green narrow‐band emission (*λ*_em_ = 481 nm, FWHM = 49 nm) with a near‐unity PLQY. Structural and photophysical investigations reveal that this high‐efficiency narrow‐band emission stems from the rigidly confined dtbp ligand, whose vibrational relaxation is effectively suppressed by the synergistic effect of metal coordination and a hydrogen‐bonding network. Remarkably, despite crystallizing in the achiral space group *Pca*2_1_ (point group *mm*2), the compound demonstrates significant chiroptical activity, exhibiting circularly polarized luminescence with a large *g*_lum_ of –0.283. This achievement represents a rare and optimal balance between ultrahigh PLQY and a substantial *g*_lum_ value. We attribute this exceptional CPL performance to the synergistic interplay of a rigid supramolecular framework and the significant lattice distortion induced by the ligand modification. This work accomplishes two key objectives: firstly, it provides a novel and effective paradigm for the simultaneous realization of narrow FWHM, high PLQY, and large *g*_lum_ in a single material. Secondly, it delivers a compelling demonstration that macroscopic chiroptical activity is not an exclusive property of chiral space groups but can be engineered through the specific arrangement of structural units in achiral crystalline systems, opening new avenues for the design of advanced CPL materials.

## Conflicts of Interest

The authors declare no conflicts of interest.

## Supporting information




**Supporting File 1**: advs74020‐sup‐0001‐SuppMat.docx.

**Supporting File 2**: advs74020‐sup‐0002‐Data.zip.

## Data Availability

The data that support the findings of this study are available from the corresponding author upon reasonable request.

## References

[1] Y. Yu , C. Wang , F.‐F. Hung , et al., “Benzo‐Extended Heli(aminoborane)s: Inner Rim BN‐Doped Helical Molecular Carbons With Remarkable Chiroptical Properties,” Journal of the American Chemical Society 146 (2024): 22600–22611, 10.1021/jacs.4c06997. 39101597

[2] X. Zhan , F.‐F. Xu , Z. Zhou , Y. Yan , J. Yao , and Y. S. Zhao , “3D Laser Displays Based on Circularly Polarized Lasing From Cholesteric Liquid Crystal Arrays,” Advanced Materials 33 (2021): 2104418, 10.1002/adma.202104418. 34337797

[3] X. Wang , B. Zhao , and J. Deng , “Liquid Crystals Doped With Chiral Fluorescent Polymer: Multi‐Color Circularly Polarized Fluorescence and Room‐Temperature Phosphorescence With High Dissymmetry Factor and Anti‐Counterfeiting Application,” Advanced Materials 35 (2023): 2304405, 10.1002/adma.202304405. 37505074

[4] Y. Zhou , H. Li , T. Zhu , T. Gao , and P. Yan , “A Highly Luminescent Chiral Tetrahedral Eu_4_L_4_(L′)_4_ Cage: Chirality Induction, Chirality Memory, and Circularly Polarized Luminescence,” Journal of the American Chemical Society 141 (2019): 19634–19643, 10.1021/jacs.9b07178. 31747264

[5] L. Tong , X. Huang , P. Wang , et al., “Stable Mid‐infrared Polarization Imaging Based on Quasi‐2D Tellurium at Room Temperature,” Nature Communications 11 (2020): 2308, 10.1038/s41467-020-16125-8. PMC7210936 32385242

[6] J. Li , Z. Yue , J. Li , et al., “Wavefront‐Controllable All‐Silicon Terahertz Meta‐Polarizer,” Science China Materials 66 (2023): 300–308, 10.1007/s40843-022-2126-0.

[7] Q. Xu , J. P. Fu , M. X. Tang , H. B. Yao , and J. Lin , “Circularly Polarized Luminescence in Chiral Materials: Navigating Trade‐Offs Between Luminescence Dissymmetry Factor and Photoluminescence Quantum Yield,” Advanced Optical Materials 13 (2025): 01569, 10.1002/adom.202501569.

[8] R. Zhang , H. Zhong , K. Yang , K. Pan , B. Zhao , and J. Deng , “Energy Transfer for Constructing Circularly Polarized Luminescence Materials: Recent Progress and Future Prospects,” Advanced Functional Materials 35 (2025): 2417308, 10.1002/adfm.202417308.

[9] J. Han , S. Guo , H. Lu , S. Liu , Q. Zhao , and W. Huang , “Recent Progress on Circularly Polarized Luminescent Materials for Organic Optoelectronic Devices,” Advanced Optical Materials 6 (2018): 1800538, 10.1002/adom.201800538.

[10] C.‐M. Shi , H. Lu , J.‐Y. Wang , G. Long , L.‐J. Xu , and Z.‐N. Chen , “Stepwise Amplification of Circularly Polarized Luminescence in Indium‐based Metal Halides by Regulating Their Structural Dimension,” Nature Communications 16 (2025): 1505, 10.1038/s41467-025-56394-9. PMC11811174 39929818

[11] Z.‐P. Yan , L. Yuan , Y. Zhang , et al., “A Chiral Dual‐Core Organoboron Structure Realizes Dual‐Channel Enhanced Ultrapure Blue Emission and Highly Efficient Circularly Polarized Electroluminescence,” Advanced Materials 34 (2022): 2204253, 10.1002/adma.202204253. 35839149

[12] H. L. Lee , S. O. Jeon , I. Kim , et al., “Multiple‐Resonance Extension and Spin‐Vibronic‐Coupling‐Based Narrowband Blue Organic Fluorescence Emitters With Over 30% Quantum Efficiency,” Advanced Materials 34 (2022): 2202464, 10.1002/adma.202202464. 35762112

[13] Y. X. Hu , J. Miao , T. Hua , et al., “Efficient Selenium‐Integrated TADF OLEDs With Reduced Roll‐Off,” Nature Photonics 16 (2022): 803–810, 10.1038/s41566-022-01083-y.

[14] Bt.2020: Parameter Values for Ultra‐High Definition Television Systems for Production And International Programme Exchange (ITU‐R, 2012), https://www.itu.int/rec/r‐rec‐bt.2020/en.

[15] Z. Zhang , Y. Lin , J. Jin , et al., “Crystalline‐Phase‐Recognition‐Induced Domino Phase Transition and Luminescence Switching for Advanced Information Encryption,” Angewandte Chemie International Edition 60 (2021): 23373–23379, 10.1002/anie.202110088. 34402142

[16] Z. Wang , Z. Zhang , L. Tao , et al., “Hybrid Chloroantimonates(III): Thermally Induced Triple‐Mode Reversible Luminescent Switching and Laser‐ Printable Rewritable Luminescent Paper,” Angewandte Chemie International Edition 58 (2019): 9974–9978. 31070295 10.1002/anie.201903945

[17] D.‐Y. Li , Y.‐B. Shang , and Q. Liu , “0D hybrid Indium Halide as a Highly Efficient X‐Ray Scintillation and Ultra‐sensitive Fluorescent Probe,” Materials Horizons 10 (2023): 5004–5015. 37642515 10.1039/d3mh00536d

[18] J. Jin , S. Geng , K. Han , Z. Xiao , and Z. Xia , “Blue‐Light‐Excitable Red‐to‐Near Infrared Photoluminescence in 0D Antimony(III) Bromide Hybrids for Supplemental Lighting,” Advanced Optical Materials 12 (2024): 2303178, 10.1002/adom.202303178.

[19] N. Lin , R.‐C. Wang , S.‐Y. Zhang , et al., “0D Hybrid Cuprous Halide as an Efficient Light Emitter and X‐Ray Scintillator,” Laser & Photonics Reviews 17 (2023): 2300427.

[20] Y.‐P. Lin , X. Lu , Z. Zhang , et al., “Organic Hybrid Tetranuclear Clusteroluminogens: Blue‐Light‐Excitable LED With Ultrahigh Luminous Efficacy,” Chemical Engineering Journal 479 (2024): 147523, 10.1016/j.cej.2023.147523.

[21] K. Zhou , B. Qi , Z. Liu , X. Wang , Y. Sun , and L. Zhang , “Advanced Organic–Inorganic Hybrid Materials for Optoelectronic Applications,” Advanced Functional Materials 34 (2024): 2411671, 10.1002/adfm.202411671.

[22] J.‐C. Jin , N.‐N. Shen , Z.‐P. Wang , Y.‐C. Peng , and X.‐Y. Huang , “Photoluminescent Ionic Metal Halides Based on s^2^ Typed Ions and Aprotic Ionic Liquid Cations,” Coordination Chemistry Reviews 448 (2021): 214185, 10.1016/j.ccr.2021.214185.

[23] L. Wang , H. Peng , Q. Wei , et al., “Finely Controlled Circularly Polarized Luminescence With Multimode Dynamic Response of Hybrid Metal Chlorides for Circularly Polarized White Light‐Emitting Diode and Programmable Information Encryption,” Laser & Photonics Reviews 19 (2025): 2400856, 10.1002/lpor.202400856.

[24] T. Song , C.‐Q. Wang , H. Lu , et al., “Achieving Strong Circularly Polarized Luminescence Through Cascade Cationic Insertion in Lead‐free Hybrid Metal Halides,” Angewandte Chemie International Edition 63 (2024): 202400769, 10.1002/anie.202400769. 38544401

[25] C. Li , Y. Wei , Y. Zhang , et al., “Efficient Ultraviolet Circularly Polarized Luminescence in Zero‐Dimensional Hybrid Cerium Bromides,” Angewandte Chemie International Edition 63 (2024): 202403727, 10.1002/anie.202403727. 38632082

[26] O. Toma , M. Allain , F. Meinardi , A. Forni , C. Botta , and N. Mercier , “Bismuth‐Based Coordination Polymers With Efficient Aggregation‐Induced Phosphorescence and Reversible Mechanochromic Luminescence,” Angewandte Chemie International Edition 55 (2016): 7998–8002, 10.1002/anie.201602602. 27166740

[27] C. Sun , Y. Li , J. Yin , et al., “Highly Stable MOF‐Type Lead Halide Luminescent Ferroelectrics,” Angewandte Chemie International Edition 63 (2024): 202407102. 10.1002/anie.202407102 38744673

[28] H. Y. Wu , C. L. Hu , M. B. Xu , et al., “From H_12_C_4_N_2_CdI_4_ to H_11_C_4_N_2_CdI_3_: A highly polarizable CdNI_3_ tetrahedron induced a sharp enhancement of second harmonic generation response and birefringence,” Chemical Science 14 (2023): 9533–9542, 10.1039/D3SC03052K. 37712033 PMC10498671

[29] F. Wang , X. Li , T. Chen , et al., “A Strategy of Chiral Cation Coordination to Achieve a Large Luminescence Dissymmetry Factor in 1D Hybrid Manganese Halides,” Chemical Science 16 (2025): 11012–11020, 10.1039/D5SC01615K. 40406220 PMC12093057

[30] J. Chen , S. Zhang , X. Pan , et al., “Structural Origin of Enhanced Circularly Polarized Luminescence in Hybrid Manganese Bromides,” Angewandte Chemie International Edition 61 (2022): 202205906. 10.1002/anie.202205906 35535865

[31] H.‐W. Lin , A. Ablez , Z.‐H. Deng , et al., “A Ligand‐Incorporating Strategy Towards Single‐Component White Light in Ionic Zero‐Dimensional Indium Chlorides,” Journal of Materials Chemistry C 12 (2024): 5184–5190, 10.1039/D3TC04659A.

[32] Y.‐Y. Ma , H.‐Q. Fu , X.‐L. Liu , et al., “Zero‐Dimensional Organic–Inorganic Hybrid Indium Chlorides With Intrinsic Blue Light Emissions,” Inorganic Chemistry 61 (2022): 8977–8981, 10.1021/acs.inorgchem.2c00518.

[33] S. Guha , J. D. Rice , Y. T. Yau , et al., “Temperature‐dependent Photoluminescence of Organic Semiconductors With Varying Backbone Conformation,” Physical Review B 67 (2003): 125204, 10.1103/PhysRevB.67.125204.

[34] R. W. Godby , M. Schluter , and L. J. Sham , “Trends in Self‐Energy Operators and Their Corresponding Exchange‐Correlation Potentials,” Physical Review B 36 (1987): 6497–6500, 10.1103/PhysRevB.36.6497. 9942359

[35] R. Terki , G. Bertrand , and H. Aourag , “Full Potential Investigations of Structural and Electronic Properties of ZrSiO_4_ ,” Microelectronic Engineering 81 (2005): 514–523, 10.1016/j.mee.2005.03.055.

[36] J. Zhou , D. Tian , W. Bai , et al., “Tunable Dimensionality and Emission of Organic Metal Halides by Denser Stacking of Pb–Br Polyhedra,” ACS Applied Materials & Interfaces 17 (2025): 19917–19927, 10.1021/acsami.4c22649. 40091168

[37] Q. Wang , W. Jiang , T.‐C. Liu , et al., “Organic Indium Halides With near‐Unity Photoluminescence Quantum Yields for Highly Efficient Luminescent Inks and White Light Emitting Diodes,” ACS Applied Materials & Interfaces 17 (2025): 24048–24057, 10.1021/acsami.4c18587. 40202305

[38] C. Sun , C.‐Q. Jing , D.‐Y. Li , et al., “In Situ Halide Vacancy Tuning of Low‐Dimensional Lead Perovskites to Realize Multiple Adjustable Luminescence Performance,” Advanced Science 12 (2025): 2412459. 40091653 10.1002/advs.202412459 PMC12079511

[39] K. Liu , A. Hou , J. Lin , et al., “High‐Stability Hybrid Antimony Halides for Thermometry in Power System Component or Circuit Monitoring,” Advanced Functional Materials 35 (2025): 2412529, 10.1002/adfm.202412529.

[40] J. Guan , Y. Zheng , P. Cheng , et al., “Free Halogen Substitution of Chiral Hybrid Metal Halides for Activating the Linear and Nonlinear Chiroptical Properties,” Journal of the American Chemical Society 145 (2023): 26833–26842, 10.1021/jacs.3c09395. 38039190

[41] Y. Deng , X. Liang , F. Li , et al., “Large Cation Engineering in Organic Antimony Halides for Low‐Loss Active Waveguide,” Laser & Photonics Reviews 17 (2023): 2300043.

[42] Y. Zhang , S. Yuan , Y. Yuan , et al., “Alleviation of π–π* Transition Enabling Enhanced Luminescence in Emerging TpyInCl x (x =3, 5) Perovskite Single Crystals,” Advanced Optical Materials 10 (2022): 2102041, 10.1002/adom.202102041.

[43] X. Zhang , X. Jiang , K. Liu , et al., “Small Organic Molecular‐Based Hybrid Halides With High Photoluminescence Quenching Temperature,” Inorganic Chemistry 61 (2022): 7560–7567, 10.1021/acs.inorgchem.2c00711. 35503095

[44] B. Su , G. Song , M. S. Molokeev , et al., “Role of Metal–Chloride Anions in Photoluminescence Regulations for Hybrid Metal Halides,” The Journal of Physical Chemistry Letters 12 (2021): 1918–1925, 10.1021/acs.jpclett.1c00182. 33591758

[45] Y.‐C. Peng , J.‐C. Jin , Q. Gu , et al., “Selective Luminescence Response of a Zero‐Dimensional Hybrid Antimony(III) Halide to Solvent Molecules: Size‐Effect and Supramolecular Interactions,” Inorganic Chemistry 60 (2021): 17837–17845, 10.1021/acs.inorgchem.1c02445. 34738796

[46] L.‐J. Xu , H. Lin , S. Lee , et al., “0D and 2D: The Cases of Phenylethylammonium Tin Bromide Hybrids,” Chemistry of Materials 32 (2020): 4692–4698, 10.1021/acs.chemmater.0c01254.

[47] S. Yakunin , B. M. Benin , Y. Shynkarenko , et al., “High‐Resolution Remote Thermometry and Thermography Using Luminescent Low‐Dimensional Tin‐Halide Perovskites,” Nature Materials 18 (2019): 846–852, 10.1038/s41563-019-0416-2. 31263225

[48] T. R. Amarante , S. Figueiredo , A. D. Lopes , I. S. Goncalves , and F. A. Almeida Paz , “4,4′‐Di‐ tert ‐butyl‐2,2′‐bipyridine,” Acta Crystallographica Section E Structure Reports Online 65 (2009): o2047, 10.1107/S1600536809029109. 21583710 PMC2977408

[49] J. Zhao , T. Zhang , X.‐Y. Dong , et al., “Circularly Polarized Luminescence From Achiral Single Crystals of Hybrid Manganese Halides,” Journal of the American Chemical Society 141 (2019): 15755–15760, 10.1021/jacs.9b08780. 31525976

[50] H. Yang , S.‐K. Peng , J. Zheng , et al., “Achiral Au(I) Cyclic Trinuclear Complexes With High‐Efficiency Circularly Polarized Near‐Infrared TADF,” Angewandte Chemie International Edition 62 (2023): 202310495, 10.1002/anie.202310495. 37638844

[51] L.‐L. Xu , H.‐F. Zhang , M. Li , et al., “Chiroptical Activity From an Achiral Biological Metal–Organic Framework,” Journal of the American Chemical Society 140 (2018): 11569–11572, 10.1021/jacs.8b06725. 30141923

[52] R. Gautier , J. M. Klingsporn , R. P. Van Duyne , and K. R. Poeppelmeier , “Optical Activity From Racemates,” Nature Materials 15 (2016): 591–592. 27088235 10.1038/nmat4628

[53] W. H. Baur and D. Kassner , “The Perils of Cc: Comparing the Frequencies of Falsely Assigned Space Groups With Their General Population,” Acta Crystallographica Section B Structural Science 48 (1992): 356–369, 10.1107/S0108768191014726.

[54] C. Sun , Y. K. Li , J. L. Yin , et al., “Highly Stable MOF‐Type Lead Halide Luminescent Ferroelectrics,” Angewandte Chemie International Edition 63 (2024): 202407102. 10.1002/anie.202407102 38744673

[55] D. S. Singh , “Structural Elucidation of Electrical and Optical Properties in Lead‐free Organic‐Inorganic Copper Bromide (CH_3_)_4_N_2_CuBr_4_ Single Crystals,” ChemistrySelect 9 (2024): 202400241.

[56] M. Wells , J. Hempel , S. Adhikari , et al., “Structure and Piezoelectricity Due to B Site Cation Variation in AB^n+^Cl_n+2_ Hybrid Histammonium Chlorometallate Materials,” Inorganic Chemistry 61 (2022): 17746–17758. 36282246 10.1021/acs.inorgchem.2c02994

[57] Y. Liu , Y.‐P. Gong , S. Geng , et al., “Hybrid Germanium Bromide Perovskites With Tunable Second Harmonic Generation,” Angewandte Chemie, International Edition 61 (2022): 202208875. 10.1002/anie.202208875 36043492

[58] H. Bouznif , F. Hajlaoui , K. Karoui , et al., “A Novel 1‐D Square‐pyramidal Coordinated Palladium (II) Hybrid Compounds C_9_H_16_N_2_PdX_4_ (X = Cl, Br) Showing Broadband Emission, Electrical Properties and Narrow Optical Band Gap,” Journal of Solid State Chemistry 131 (2022): 123149.

[59] M. Essid , Z. Aloui , V. Ferretti , et al., “ _6_ ,” Inorganica Chimica Acta 457 (2017): 122–129, 10.1016/j.ica.2016.12.012.

[60] X. Han , P. Cheng , S. Han , et al., “Multi‐stimuli‐responsive Luminescence Enabled by Crown Ether Anchored Chiral Antimony Halide Phosphors,” Chemical Science 15 (2024): 3530–3538, 10.1039/D3SC06362C. 38455020 PMC10915841

[61] C.‐Y. Chai , C.‐D. Liu , B.‐D. Liang , X.‐B. Han , W. Zhang , and C.‐C. Fan , “Circularly Polarized Luminescence in Zero‐Dimensional Antimony Halides: Structural Distortion Controlled Luminescence Thermometer,” The Journal of Physical Chemistry Letters 14 (2023): 4063–4070, 10.1021/acs.jpclett.3c00693. 37094225

[62] H.‐L. Xuan , J.‐L. Li , L.‐J. Xu , D.‐S. Zheng , and Z.‐N. Chen , “Circularly Polarized Luminescence based on 0D Lead‐Free Antimony (III) Halide Hybrids,” Advanced Optical Materials 10 (2022): 2200591.

