# Supplementary material for: Simultaneously Achieving Efficient Narrow‐Band Emission and Large Emission Dissymmetry Factor in an Achiral Hybrid Indium Chloride
Source: Adv Sci (Weinh). 2026 Jan 22;13(19):e22728. doi: 10.1002/advs.202522728 (PMC13045219; doi:10.1002/advs.202522728)
Supplement: Supplementary file 1 — Supporting File 1: advs74020‐sup‐0001‐SuppMat.docx. [file ADVS-13-e22728-s002.docx]

Supporting information

Simultaneously Achieving Efficient Narrow-Band Emission and Large Emission Dissymmetry Factor in an Achiral Hybrid Indium Chloride

Haowei Lin, ^[a,b,c]^, Abdusalam Ablez, ^[a,b,d]^, Xinping Guo, ^[a,b,d]^, Yingchen Peng, ^[a]^, Jiance Jin, ^[a]^, Zhihua Chen, ^[a,b,c]^, Kezhao Du, *^,[e]^, Zeping Wang, *^,[a]^, Xiaoying Huang, *^,[a,b,c]^

[a] H. Lin, A. Ablez, X. Guo, Y. Peng, J. Jin, Z. Chen, Dr. Z. Wang, Prof. Dr. X. Huang
State Key Laboratory of Structural Chemistry
Fujian Institute of Research on the Structure of Matter
The Chinese Academy of Sciences, Fuzhou, Fujian, 350002 (P. R. China)
E-mail: [xyhuang@fjirsm.ac.cn](mailto:xyhuang@fjirsm.ac.cn)`

[b] A. Ablez, X. Guo
Fujian College
University of Chinese Academy of Sciences, Fuzhou, Fujian 350002 (P. R. China)

[c] H. Lin, Z. Chen, Prof. Dr. X. Huang

University of Chinese Academy of Sciences, Beijing 100049 (P. R. China)

[d] A. Ablez, X. Guo
College of Chemistry
Fuzhou University, Fuzhou, Fujian, 350007 (P. R. China)

[e] Prof. Dr. K Du

College of Chemistry and Materials Science, Fujian Provincial Key Laboratory of Advanced Materials Oriented Chemical Engineering,

Fujian Normal University, Fuzhou 350007 (P. R. China)

Supporting information for this article is given via a link at the end of the document.

**Abstract:** To achieve high contrast in three-dimensional display, circularly polarized luminescence (CPL) materials should simultaneously have narrow full width at half-maximum (FWHM), high photoluminescence quantum yield (PLQY), and large dissymmetry factor (*g*_lum_). However, very few materials can fulfill it. Herein, we report a zero-dimensional hybrid indium chloride [BPy][InCl_4_(dtbp)] (BPy = *N*-butylpyridinium, dtbp = 4,4’-di-tert-butyl-2,2’-dipyridyl). Although crystallizing in the achiral point group *mm*2, the compound demonstrates remarkable chiroptical activity. It shows efficient blue-green emission peaking at 481 nm with a FWHM of 49 nm. The coordination with In^3+^ in the inorganic unit, combined with abundant intermolecular interactions, effectively suppresses vibrational relaxation of the organic ligand dtbp, resulting in narrow-band emission. Moreover, owing to the synergy between this structural rigidity and the distortion of the InCl_4_(dtbp) unit, this compound overcomes the trade-off between PLQY and *g*_lum_, exhibiting a PLQY approaching 100% and a |*g*_lum_| value of 0.283. Photophysical characterizations and density-functional theory calculations indicate that the high-efficiency emission originates from the ligand. As a rare example of optically active achiral crystals, this compound not only offers a promising candidate for CPL materials integrating narrow FWHM, high PLQY, and large *g*_lum_, but also provides valuable insight into the chiroptical properties of achiral crystalline systems.

Table of Contents

| **Experimental Section** | 3 |
| --- | --- |
| Materials. | 4 |
| Synthesis of [BPy][InCl_4_(dtbp)]. | 4 |
| Computational details. | 4 |
| **Table S1.** Crystallographic data and refinement details for [BPy][InCl_4_(dtbp)]. | 6 |
| **Table S2.** Selected bond lengths (Å) and bond angles (°) for [BPy][InCl_4_(dtbp)]. | 7 |
| **Figure S1**. *ORTEP* drawing (30% ellipsoid probability) of the asymmetric unit of [BPy][InCl_4_(dtbp)] at 293 K. Hydrogen atoms have been omitted for clarity; the structure contains a group of disordered carbon atoms, with the bonds they form indicated by dashed lines. | 7 |
| **Figure S2**. Schematic diagram of the hydrogen-bonded supramolecular network of [BPy][InCl_4_(dtbp)] viewed along the *a*, *b*, and *c* axis, respectively. | 8 |
| **Table S3.** Hydrogen bonds for [BPy][InCl_4_(dtbp)]. | 9 |
| **Figure S3**. Comparison of experimental PXRD pattern of [BPy][InCl_4_(dtbp)] with simulated pattern of SCXRD data. | 9 |
| **Figure S4**. The TG curve for [BPy][InCl_4_(dtbp)]. | 10 |
| **Figure S5**. PL lifetime of [BPy][InCl_4_(dtbp)] at 300 K. | 10 |
| **Figure S6.** (a) Electron distribution map on the highest occupied molecular orbital (HOMO) of dtbp. (b) Electron distribution map on the lowest occupied molecular orbital (LUMO) of dtbp. | 11 |
| **Figure S7.** The electronic band structure of [BPy][InCl_4_(dtbp)]. | 11 |
| **Figure S8.** Tauc plot showing the experimental indirect band gap of [BPy][InCl_4_(dtbp)]. | 12 |
| **Table S4**. Study on the relationship between PLQY and FWHM for different 0-D hybrid main-group metal halides. | 13 |
| **Figure S9**. PL excitation and emission spectra of dtbp at 300 K. | 14 |
| **Figure S10**. PL lifetime of dtbp at 300 K. | 14 |
| **Table S5.** Crystallographic data for dtbp. | 15 |
| **Figure S11.** Simulated energy frames representing the total energy topology in [BPy][InCl_4_(dtbp)] (a-c) and B (d-f), viewed along the *a*, *b,* and *c* axis, respectively. | 16 |
| **Table S6.** The calculated interaction energy (*E*_int_). | 16 |
| **Table S7.** PL and CPL properties of OIMHs with non-chiral *mm*2 point groups. | 17 |
| **Table S8.** Comparison of PLQY, *g*_lum_, and FWHM of some reported 0-D OIMHs with the present work. | 18 |
| **References** | 18 |

Experimental Procedures

Single crystal X-ray diffraction data were collected with graphite-monochromated CuK*α* (*λ* = 1.54178 Å) using an XtaLAB Synergy R, HyPix diffractometer at 293(2) K. Powder X-ray diffraction (PXRD) patterns were performed on a Rigaku Miniflex-II diffractometer with Cu Kα radiation (*λ* = 1.54178 Å) in the angular range of 2*θ* = 5 - 65°. The simulated PXRD pattern is calculated from the SCXRD data using the Mercury program. Thermogravimetric (TG) analysis was performed on a NETZSCH STA 449F3 unit at a heating rate of 10 K min^-1^ under a nitrogen atmosphere. Solid-state optical diffuse reflectance spectra were measured on a Shimadzu 2600 UV/vis spectrometer at room temperature (RT) in the 800 - 200 nm range. A BaSO_4_ plate is utilized as a standard with 100% reflectance. The absorption data were obtained by converting the diffuse reflection spectrum through the Kubelka–Munk function.^[1]^ Photoluminescence excitation (PLE) spectra, photoluminescence (PL) spectra, time-resolved PL spectra, and PLQYs were recorded on an Edinburgh FLS1000 NV/V/NIR fluorescence spectrometer. The femtosecond transient absorption (fs-TA) spectroscopy setup is powered by a Ti: sapphire laser amplifier (Coherent Astrella-Tunalbe-USP, USA), which can generate 800 nm pulse train with a temporal pulse width of 35 fs, pulse energy of 6.1 mJ, and repetition frequency of 1000 Hz. The fundamental beam was split into two branches. A branch of the fundamental beam was attenuated and focused into a sapphire crystal to generate the broadband probe pulses in the visible region. The frequency of another branch of the fundamental beam was doubled to 400 nm by a BBO crystal. The pump pulses (400 nm) were then chopped at the frequency of 500 Hz by an optical chopper and purified by a bandpass (400 ± 10 nm). The pump pulse energy was attenuated by neutral-density filter wheels. The time delay between pump and probe pulses was controlled by a motorized translation stage. The pump and probe pulses were focused on the same spot of the single crystal sample, which is fixed on a double-sided polished alumina substrate. The pump's focal size was intentionally adjusted to be much greater than that of the probe so that the excitation density in the probing area was homogeneous. The transmitted probe pulses were sent into the visible spectrometers. The sample was stable, and no precipitate had appeared during the experiment. Spectrum or kinetics changes due to photodegradation were not observed based on scan-to-scan comparisons. Solid-state circular dichroism (CD) spectra were acquired using a JASCO J-1500 CD spectrometer with an attachment of an integrating sphere. The sample was ground into powder. Circularly polarized luminescence (CPL) spectra were collected using a JASCO CPL-300 spectrometer.

**Materials.** All purchased reagents were utilized directly without further purification. The detailed information for the reagents is listed as follows: 1-butylpyridinium chloride ([BPy]Cl, 99%, Lanzhou Greenchem ILs, Lanzhou, China); 4,4’di-tert-butyl-2,2’-dipyridyl (dtbp, 98%, Adamas, Shanghai, China); indium (III) chloride tetrahydrate (InCl_3_·4H_2_O, 99.99%, 9dingchem, Shanghai, China); acetonitrile (CH_3_CN, AR, Sinopharm Chemical Reagent Co., Ltd., Shanghai, China).

**Synthesis of [BPy][InCl_4_(dtbp)].** [BPy][InCl_4_(dtbp)] was synthesized by the solvothermal process. Firstly, the mixture of InCl_3_·4H_2_O (0.5 mmol, 0.147 g), dtbp (0.5 mmol, 0.134 g), [BPy]Cl (0.5 mmol, 0.0855 g), and CH_3_CN (5 mL) was added into a 20 mL glass bottle, which resulted in the formation of a white precipitate and a cloudy liquid in the glass bottle. The vial was sonicated for 5 minutes to ensure thorough mixing, and then heated in an oven at 80 °C for 10 minutes. After heating, the initially turbid mixture turned into a light green clear solution. Once the solution was cooled to room temperature, the vial was sealed with parafilm, and several small holes were punctured in the film using a needle to allow for controlled solvent evaporation. The solution was left at room temperature for 3 days to obtain colorless lumpy crystals. It was washed two to three times with acetonitrile and dried naturally in air. The yield was calculated to be nearly 83.3% based on In atom. EA: Calcd (%): C, 49.04; H, 5.79; N, 6.35. Found (%): C, 49.07; H, 5.78; N, 6.30%.

***Computational details*.**

According to the single-crystal structure refinement results, DFT calculations of [BPy][InCl_4_(dtbp)] were implemented in the Vienna ab initio simulation package (VASP).^[2]^ The generalized gradient approximation (GGA) for the exchange-correlation term with the Perdew-Burke-Ernzerhof (PBE) exchange-correlation functional was applied for electron-electron exchange correlation processes.^[3]^ Projected augmented wave (PAW) potentials were used with the valence states 2s and 2p for C and N, 5s and 5p for In, 3s and 3p for Cl. To ensure sufficient accuracy, the cut-off energy of 500 eV for the plane wave expansion was chosen, self-consistent field (SCF) computations were set to a convergence criterion of 1 × 10^−5^ eV, and the force criterion was 0.02 eV Å^−1^. CrystalExplorer 17 was used to evaluate and visualize the pairwise interaction energies of [BPy][InCl_4_(dtbp)] crystal.^[4]^ The calculations are based on the B3LYP/6-31G(d.p) molecular wavefunctions. The energy components calculated within this method are electrostatic, polarization, dispersion, and exchange-repulsion, and finally the total interaction energy, where *E*_total_ = 1.057 *E*_ele_ + 0.740 *E*_pol_ + 0.871 *E*_disp_ + 0.618 *E*_rep_. Pairwise interaction energies between molecules were calculated considering a radius of 3.8 Å from the centroid of a molecule to an atom (of another molecule) belonging to its nearest neighbor. The tube size used in all the energy frameworks was 50, and the lower energy threshold (cutoff) value was set to 2.0.

Results and Discussion

Table S1. Crystallographic data and refinement details for [BPy][InCl_4_(dtbp)].

| Compound | [BPy][InCl_4_(dtbp)] |
| --- | --- |
| CCDC number | 2344052 |
| Empirical formula | C_27_H_38_Cl_4_InN_3_ |
| Formula Mass | 661.22 |
| Crystal system | Orthorhombic |
| Space group | *Pca*2_1_ |
| *a*/Å | 12.72960(10) |
| *b*/Å | 11.8279(2) |
| *c*/Å | 20.5592(3) |
| *α*/° | 90 |
| *β*/° | 90 |
| *γ*/° | 90 |
| *V*/Å^3^ | 3095.48(7) |
| *Z* | 4 |
| *T*/K | 293(2) |
| *λ*/Å | 1.54178 |
| *F*(000) | 1352 |
| *ρ*_calcd_/g cm^-3^ | 1.419 |
| *μ*/mm^-1^ | 9.424 |
| Measured refls. | 15952 |
| Independent refls. | 5348 |
| No. of parameters | 370 |
| *R*_int_ | 0.0319 |
| *R*_1_ (*I* > 2*σ*(*I*))^a^ | 0.0254 |
| *wR*(*F*^2^) (*I* > 2*σ*(*I*))^b^ | 0.0625 |
| *GOF* | 1.058 |

[a] *R*_1_ = ∑║*F*_o_│–│*F*_c_║/∑│*F*_o_│, [b] *wR*_2_ = [ ∑*w*(*F*_o_^2^−*F*_c_^2^)^2^/∑*w*(*F*_o_^2^)^2^]^1/2^.

Table S2. Selected bond lengths (Å) and bond angles (°) for [BPy][InCl_4_(dtbp)].

| In(1)-N(2) | 2.307(4) | In(1)-Cl(1) | 2.5129(10) |
| --- | --- | --- | --- |
| In(1)-N(1) | 2.310(4) | In(1)-Cl(4) | 2.5114(12) |
| In(1)-Cl(3) | 2.4431(12) | In(1)-Cl(2) | 2.4441(13) |
|  |  |  |  |
| N(2)-In(1)-N(1) | 71.39(14) | Cl(1)-In(1)-Cl(4) | 166.13(7) |
| N(1)-In(1)-Cl(1) | 84.30(11) | N(2)-In(1)-Cl(1) | 85.87(10) |
| N(1)-In(1)-Cl(2) | 95.85(5) | N(2)-In(1)-Cl(2) | 94.23(10) |
| N(1)-In(1)-Cl(3) | 94.70(10) | N(2)-In(1)-Cl(3) | 165.84(10) |
| N(1)-In(1)-Cl(4) | 83.32(11) | N(2)-In(1)-Cl(4) | 84.28(11) |


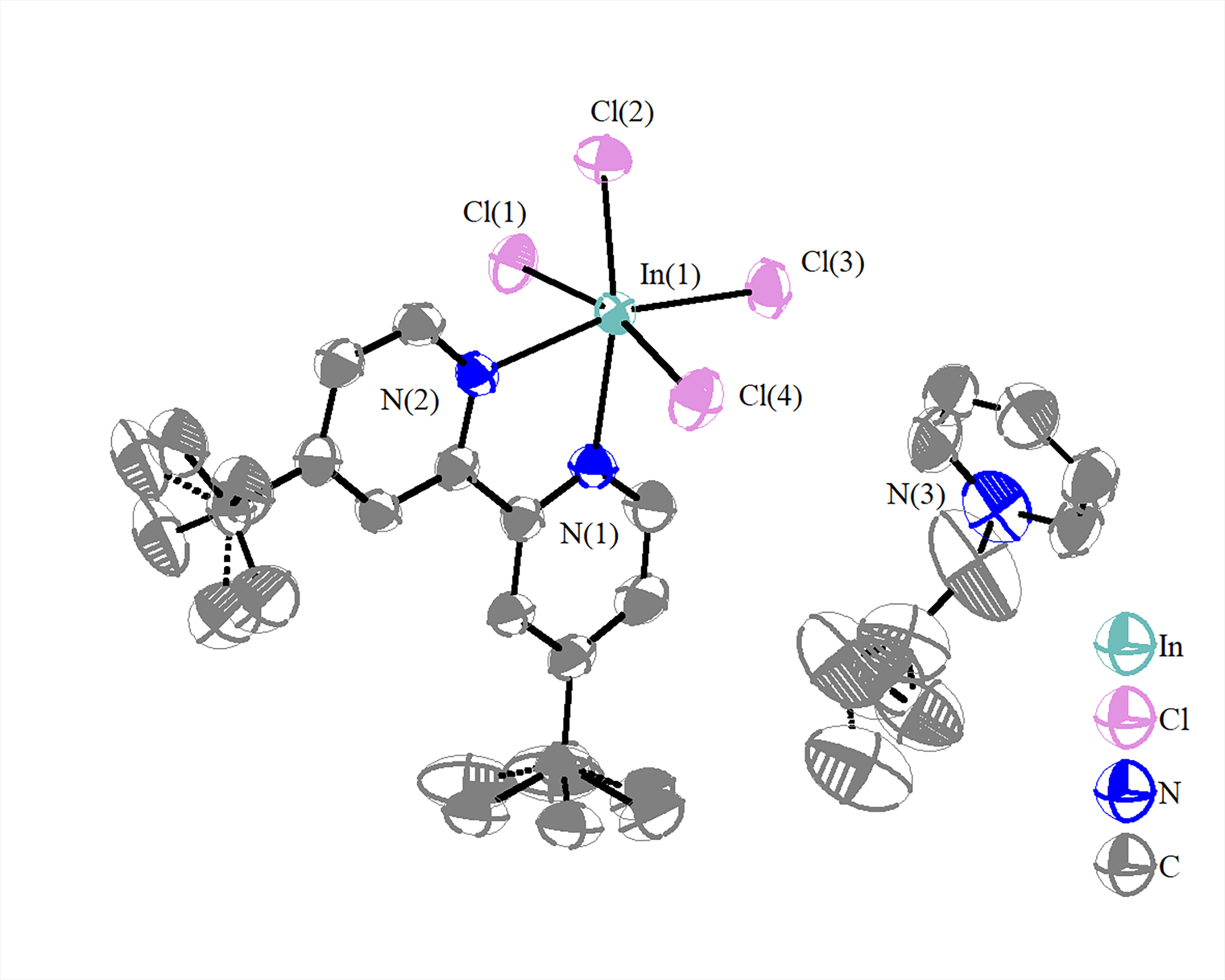


Figure S1. *ORTEP* drawing (30% ellipsoid probability) of the asymmetric unit of [BPy][InCl_4_(dtbp)] at 293 K. Hydrogen atoms have been omitted for clarity; the structure contains a group of disordered carbon atoms, with the bonds they form indicated by dashed lines.


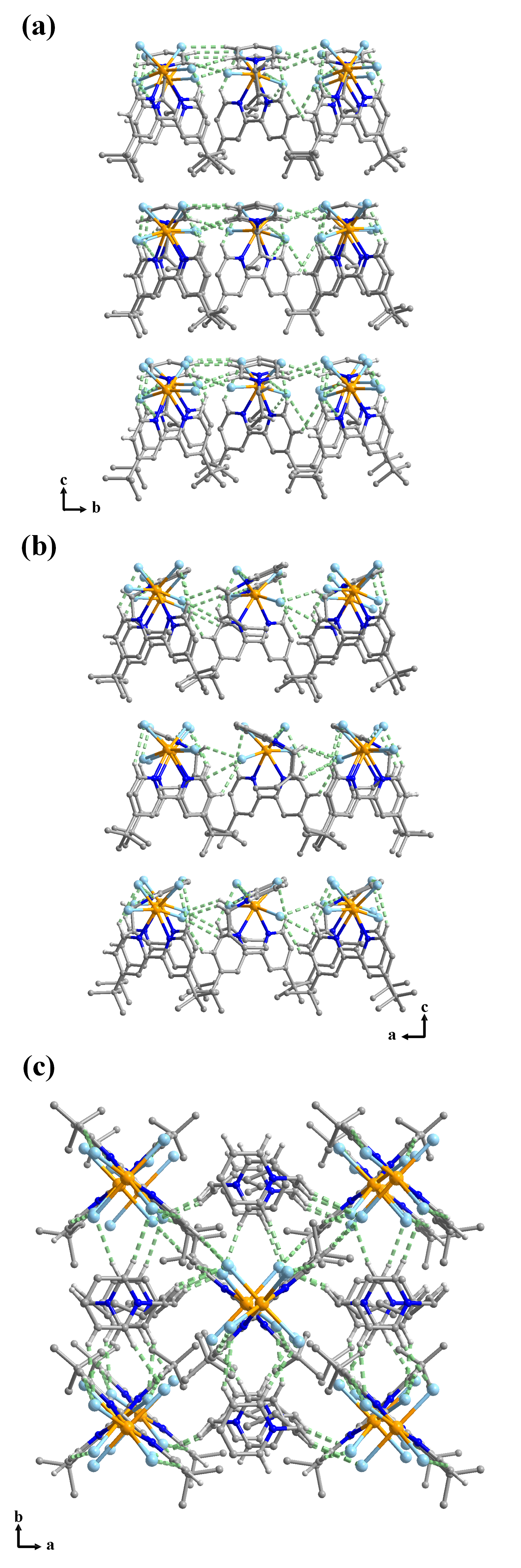


Figure S2. Diagrams of the hydrogen-bonded supramolecular network of [BPy][InCl_4_(dtbp)] viewed along the *a*, *b,* and *c* axis, respectively.

Table S3. Hydrogen bonds for [BPy][InCl_4_(dtbp)].

| D-H···A | *d*(D-H) | *d*(H···A) | *d*(D···A) | <(DHA) |
| --- | --- | --- | --- | --- |
| **anion-anion H-bond** |  |  |  |  |
| C(1)-H(1A)···Cl(3) | 0.93 | 2.86 | 3.496(5) | 127.0 |
| C(2)-H(2A)···Cl(4)#1 | 0.93 | 2.98 | 3.697(5) | 134.8 |
| C(10)-H(10A)···Cl(2) | 0.93 | 2.84 | 3.489(5) | 127.4 |
| **anion-cation H-bond** |  |  |  |  |
| C(19)-H(19A)···Cl(1)#2 | 0.93 | 2.64 | 3.566(6) | 172.3 |
| C(20)-H(20A)···Cl(2)#2 | 0.93 | 2.87 | 3.663(7) | 143.9 |
| C(23)-H(23C)···Cl(3) | 0.93 | 2.76 | 3.600(7) | 150.6 |
| C(24)-H(23B)···Cl(4)#1 | 0.97 | 2.77 | 3.512(8) | 133.9 |
| C(25)-H(25C)···Cl(4)#1 | 0.97 | 2.86 | 3.629(9) | 136.4 |
| Symmetry transformations used to generate equivalent atoms: #1 -1/2+*x*, 1-*y*, +*z*, #2 +*x*, 1+*y*, +*z* | | | | |


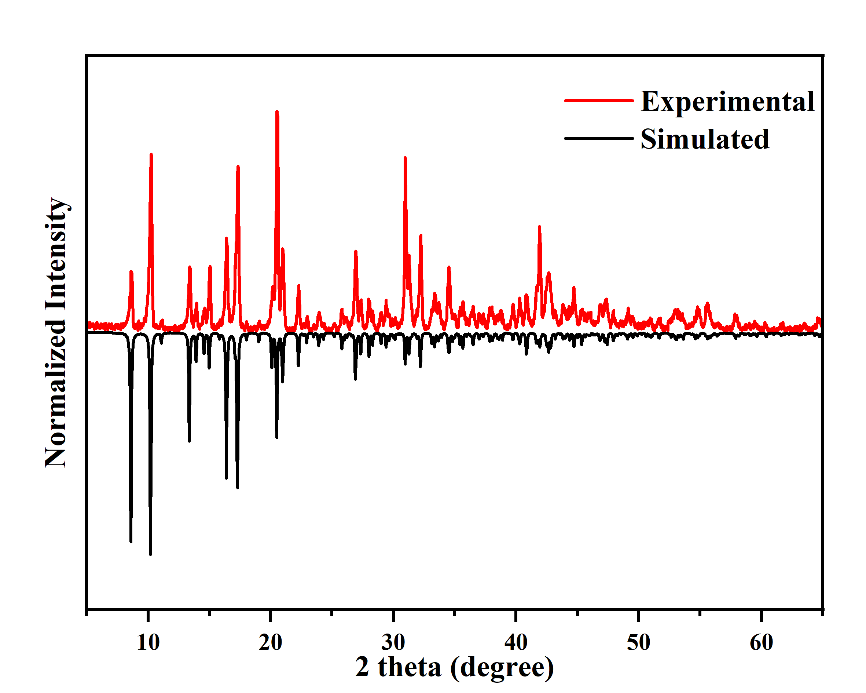


Figure S3. Comparison of experimental PXRD pattern of [BPy][InCl_4_(dtbp)] with the simulated pattern from SCXRD data.


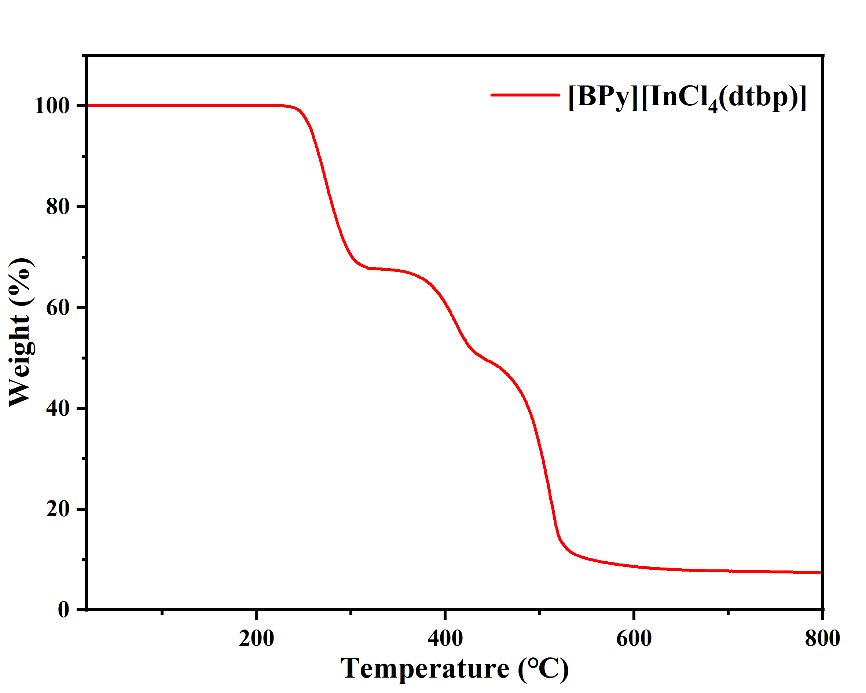


Figure S4. The TG curve for [BPy][InCl_4_(dtbp)].


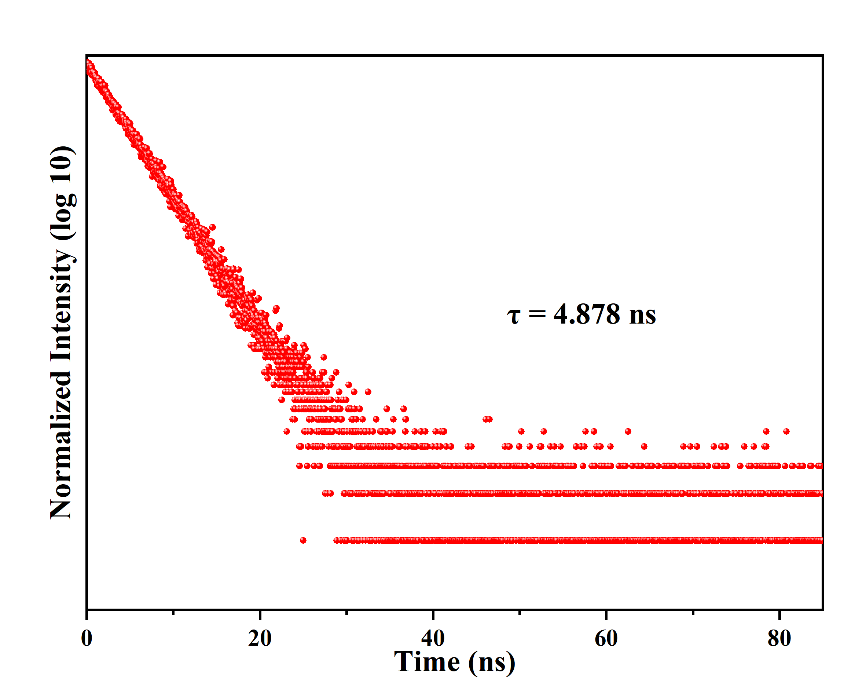


Figure S5. PL lifetime of [BPy][InCl_4_(dtbp)] at 300 K.


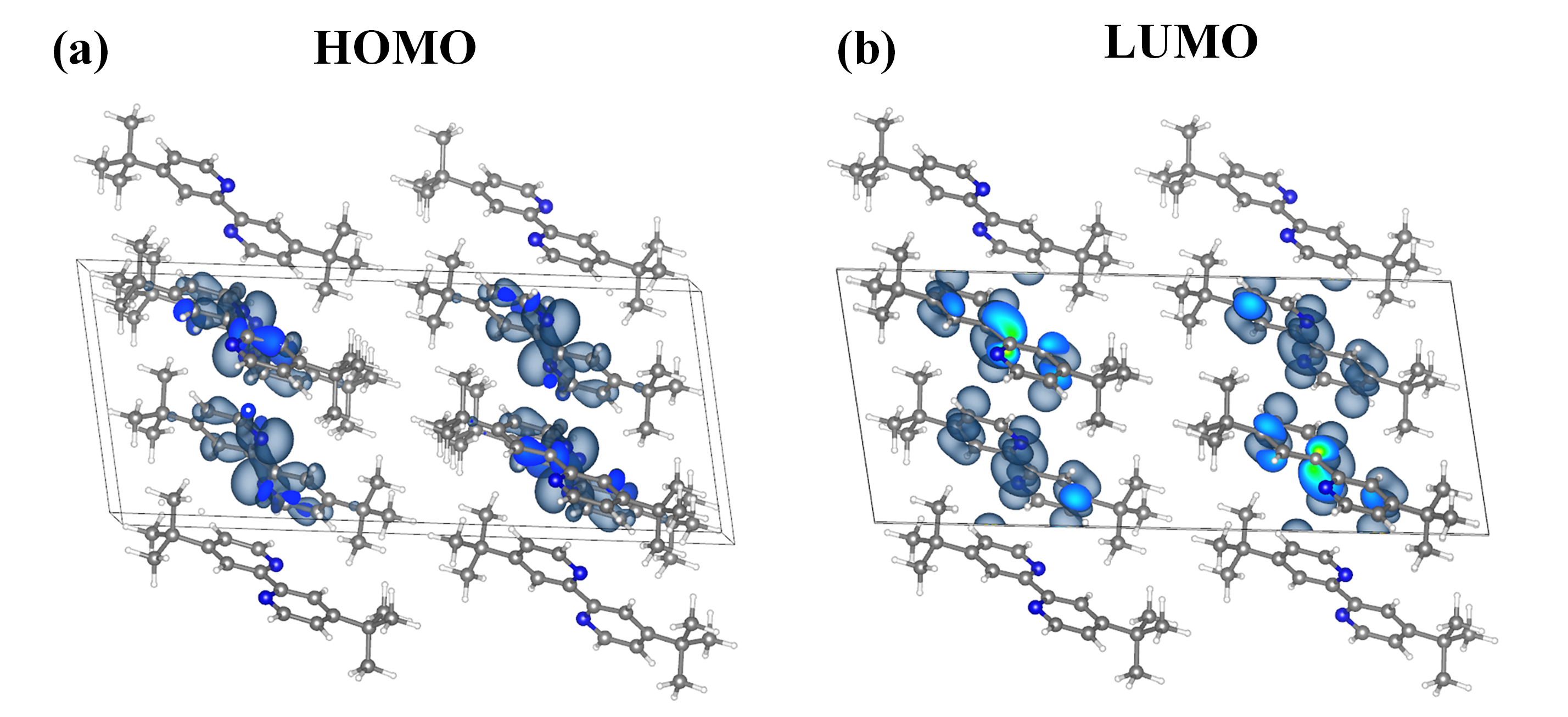


Figure S6. (a) Electron distribution map on the highest occupied molecular orbital (HOMO) of dtbp. (b) Electron distribution map on the lowest occupied molecular orbital (LUMO) of dtbp.


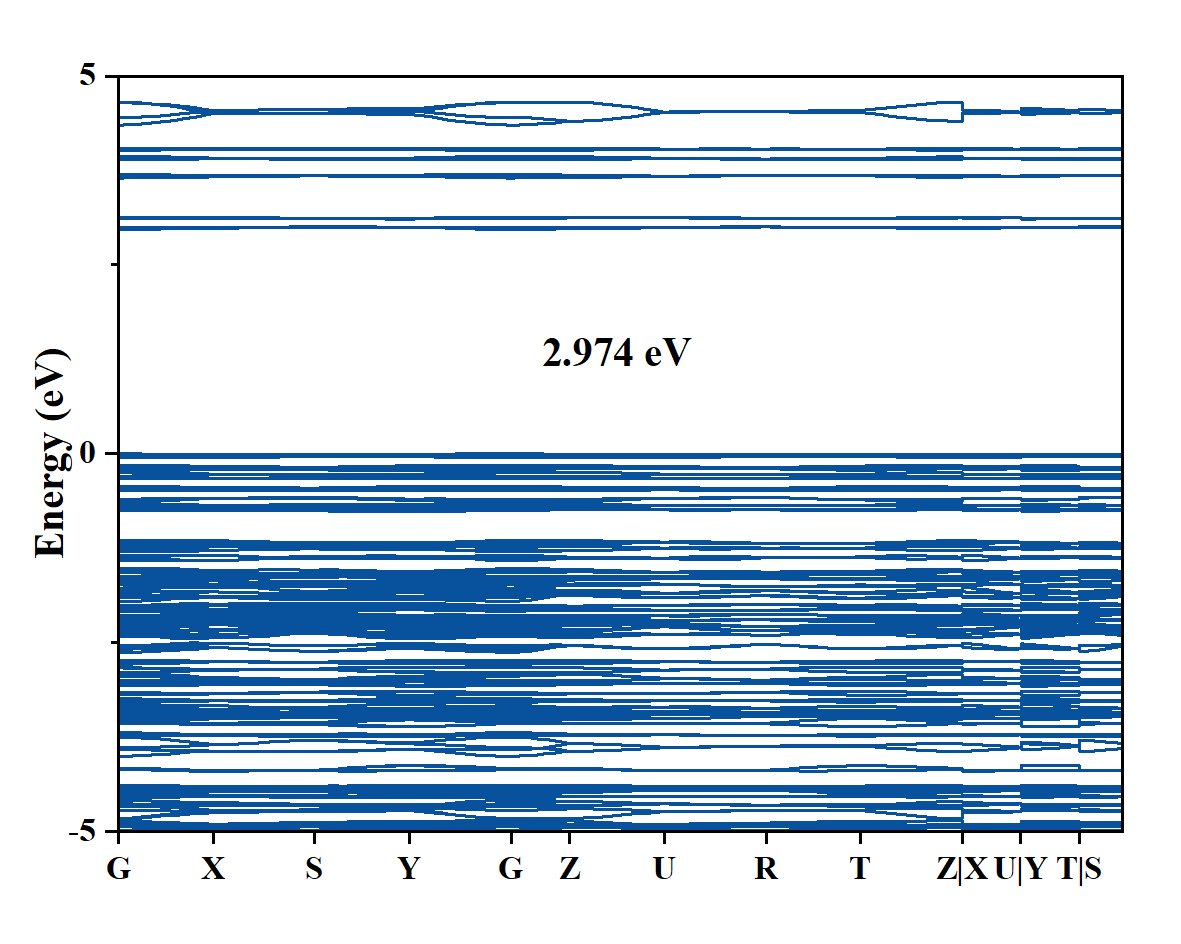


Figure S7. The electronic band structure of [BPy][InCl_4_(dtbp)].


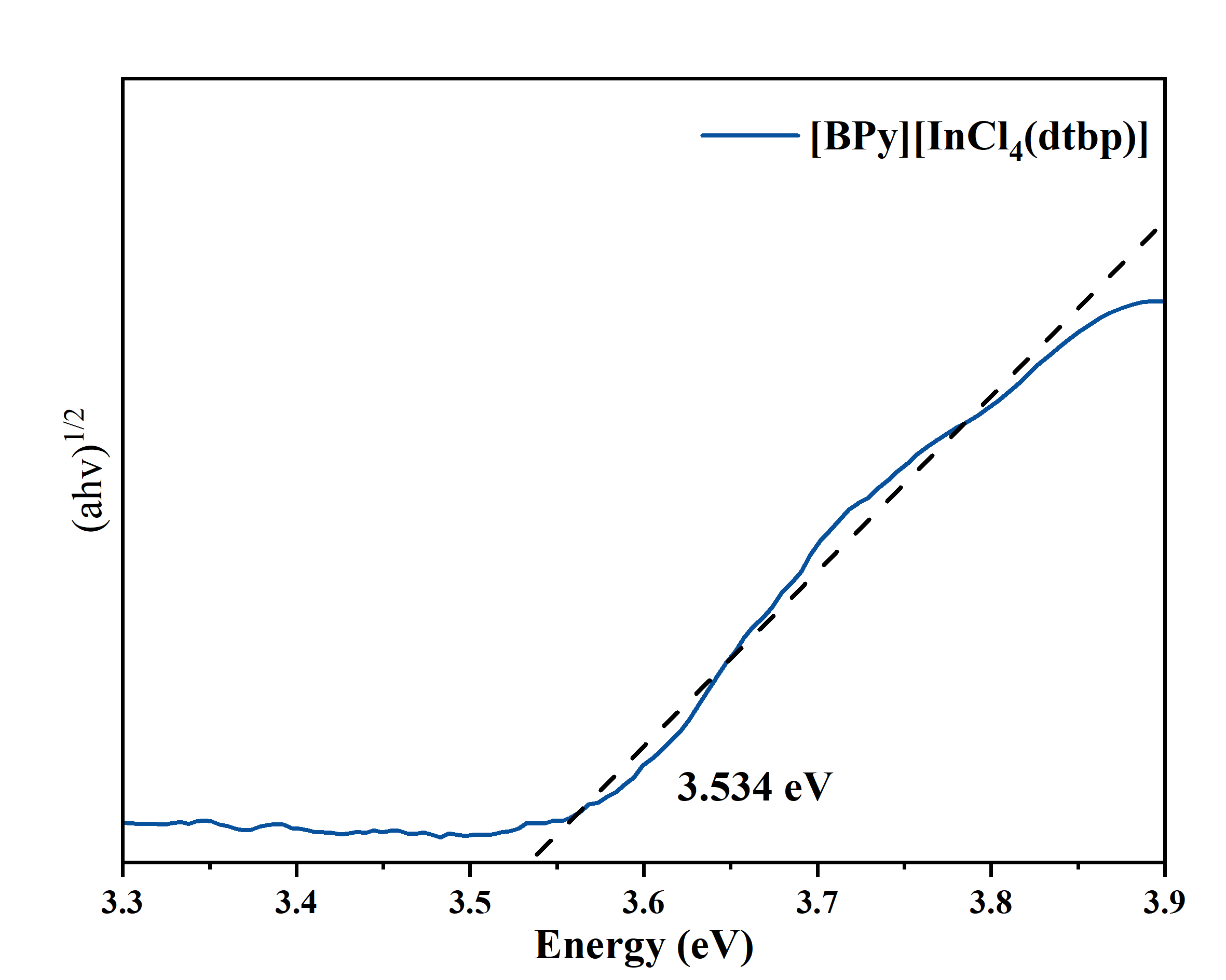


Figure S8. Tauc plot showing the experimental indirect band gap of [BPy][InCl_4_(dtbp)].

Table S4. Study on the relationship between PLQY and FWHM for different 0-D hybrid main-group metal halides.

| Compound | PLQY (%) | FWHM (nm) | Ref. |
| --- | --- | --- | --- |
| [BPy][InCl_4_(dtbp)] | 100 | 49 | This work |
| [(PEA)_4_SnBr_6_][(PEA)Br]_2_[CCl_2_H_2_]_2_ | 89.5 | 112 | ^[5]^ |
| (C_4_N_2_H_14_I)_4_SnI_6_ | 75 | 125 | ^[6]^ |
| (C_4_N_2_H_14_Br)_4_SnBr_6_ | 100 | 107 | ^[6]^ |
| TpyInCl_5_ | 47.66 | 46 | ^[7]^ |
| (C_2_H_8_N)_4_InCl_7_ | 13.9 | 76 | ^[8]^ |
| [H_2_EP]_2_InCl6·Cl·H_2_O·C_3_H_6_O | 13.44 | 37 | ^[9]^ |
| [H_3_AEP]InCl_6_·H_2_O | 4.12 | 77 | ^[9]^ |
| [D(t-Bu)EDA]_2_(InBr_6_)(InBr_4_(H_2_O)_2_) | 98.26 | 127 | ^[10]^ |
| DMA_4_[InCl_6_]Br | 81.4 | 140 | ^[11]^ |
| [Ammim][InCl_4_(dmbp)] | 16.7 | 197 | ^[12]^ |
| (C_6_N_2_H_10_)_2_PbBr_6_ | 35.4 | 209 | ^[13]^ |
| W-[DADPA]PbBr_5_ | 3.7 | 134 | ^[14]^ |
| B-[DADPA]PbBr_5_ | 38.1 | 31 | ^[14]^ |
| G-[DADPA]PbBr_5_ | 80.8 | 27 | ^[14]^ |
| [EtPPh_3_]_2_[SbCl_5_] | 90 | 123 | ^[15]^ |
| (TMA)_2_SbCl_5_ | 72.1 | 154 | ^[16]^ |
| (TMAA)_2_SbCl_5_ | 98.3 | 163 | ^[16]^ |
| (C_10_H_22_N)_6_SbBr_9_·H_2_O | 30 | 146 | ^[17]^ |
| (EnrofloH_2_)BiCl_5_·Cl·2(H_2_O)·H_3_O | 9.14 | 37 | ^[18]^ |

PEA = phenylethylammonium; C_4_N_2_H_14_ = *N*,*N*'-dimethylethylenediamine-1,2-diammonium; Tpy = di-protonated 2,2′:6′,2″-tripyridine; C_2_H_8_N = dimethylammonium; EP = protonated 1-ethylpiperazine; AEP = 1-(2-aminoethyl)piperazin-1-ium; D(t-Bu)EDA = *N*,*N*′-ditert-butylethylenediammonium; DMA = dimethylammonium; Ammim = 1-ally-2,3-dimethylimidazolium; dmbp = 4,4'-dimethyl-2,2'-bipyridyl; C_6_N_2_H_10_ = 3-(aminomethyl)pyridinium; DADPA = diaminodipropylammonium; EtPPh_3_ = ethyltriphenylphosphonium; TMA = tetramethylammonium; TMAA = *N*,*N*,*N*-trimethyltrimethyl-1-adamantylammonium; C_10_H_22_N = 4-(tert-buty)cyclohexanammonium; EnrofloH_2_ = enrofloxacinium


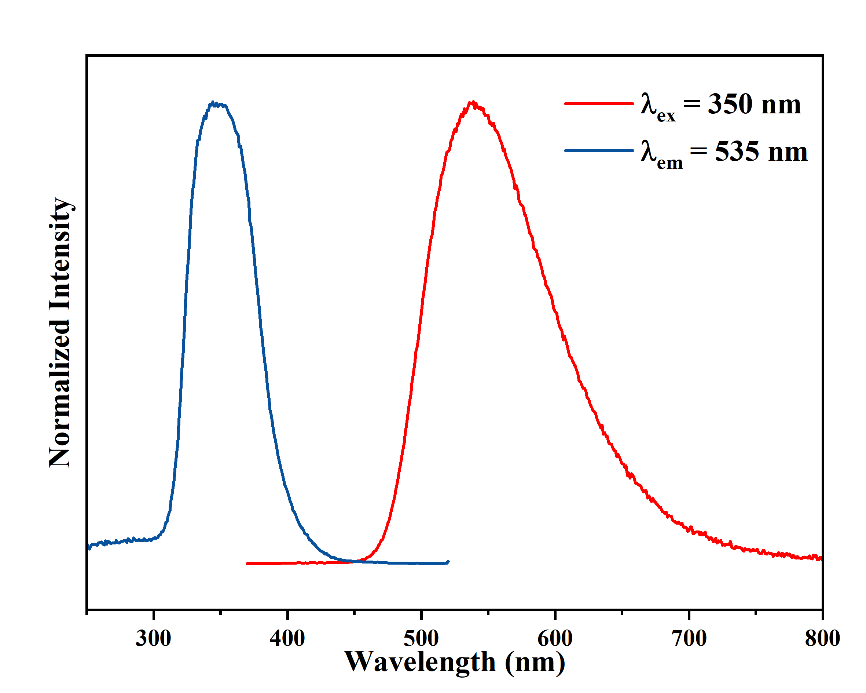


Figure S9. PL excitation and emission spectra of dtbp at 300 K.


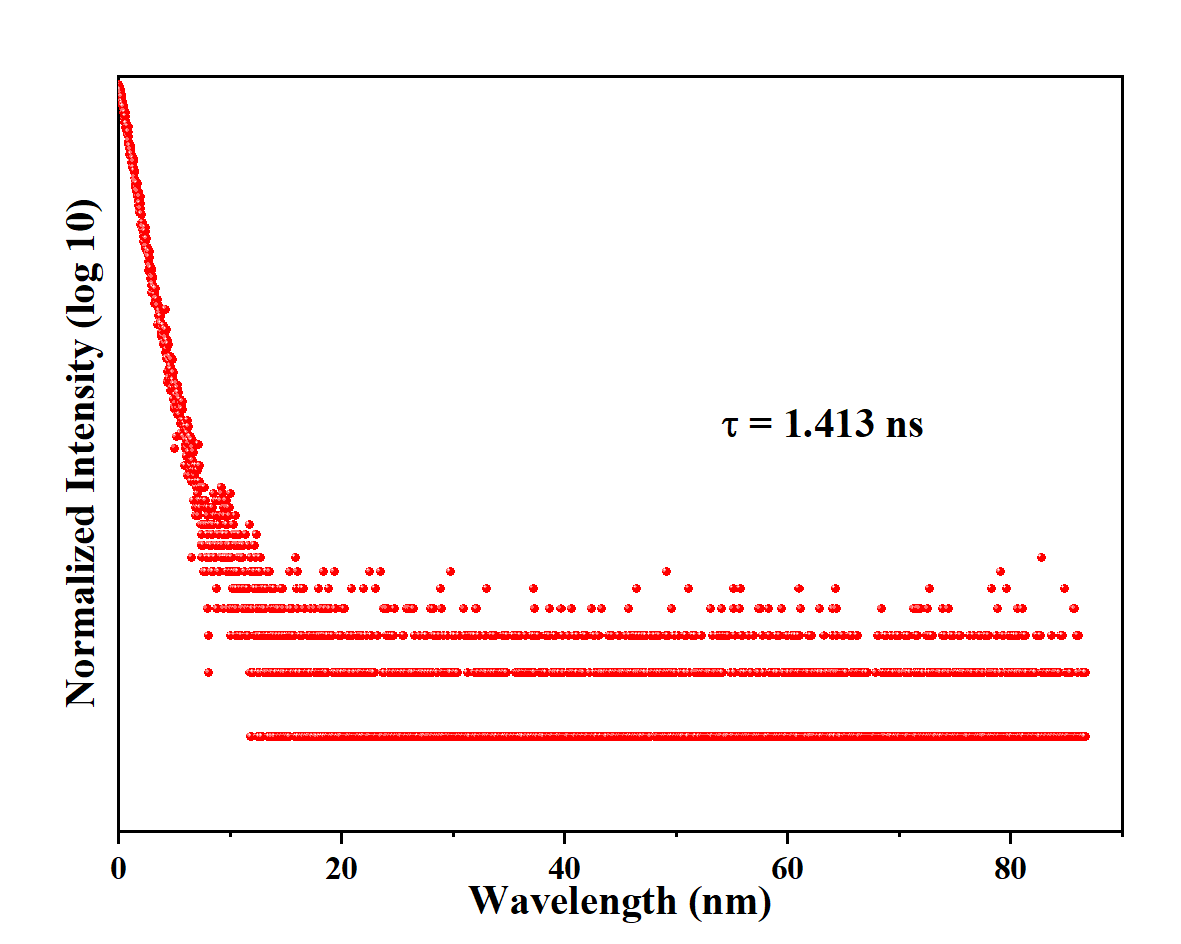


Figure S10. PL lifetime of dtbp at 300 K.

Table S5. Crystallographic data for dtbp.^[19]^

| Compound | dtbp |
| --- | --- |
| CCDC number | 744523 |
| Empirical formula | C_18_H_24_N_2_ |
| Formula Mass | 268.39 |
| Crystal system | Monoclinic |
| Space group | *P*2_1_/*c* |
| *a*/Å | 10.241(5) |
| *b*/Å | 6.228(3) |
| *c*/Å | 24.559(10) |
| *β*/° | 99.75(3) |
| *V*/Å^3^ | 1543.7(12) |
| *Z* | 4 |
| *T*/K | 296(2) |


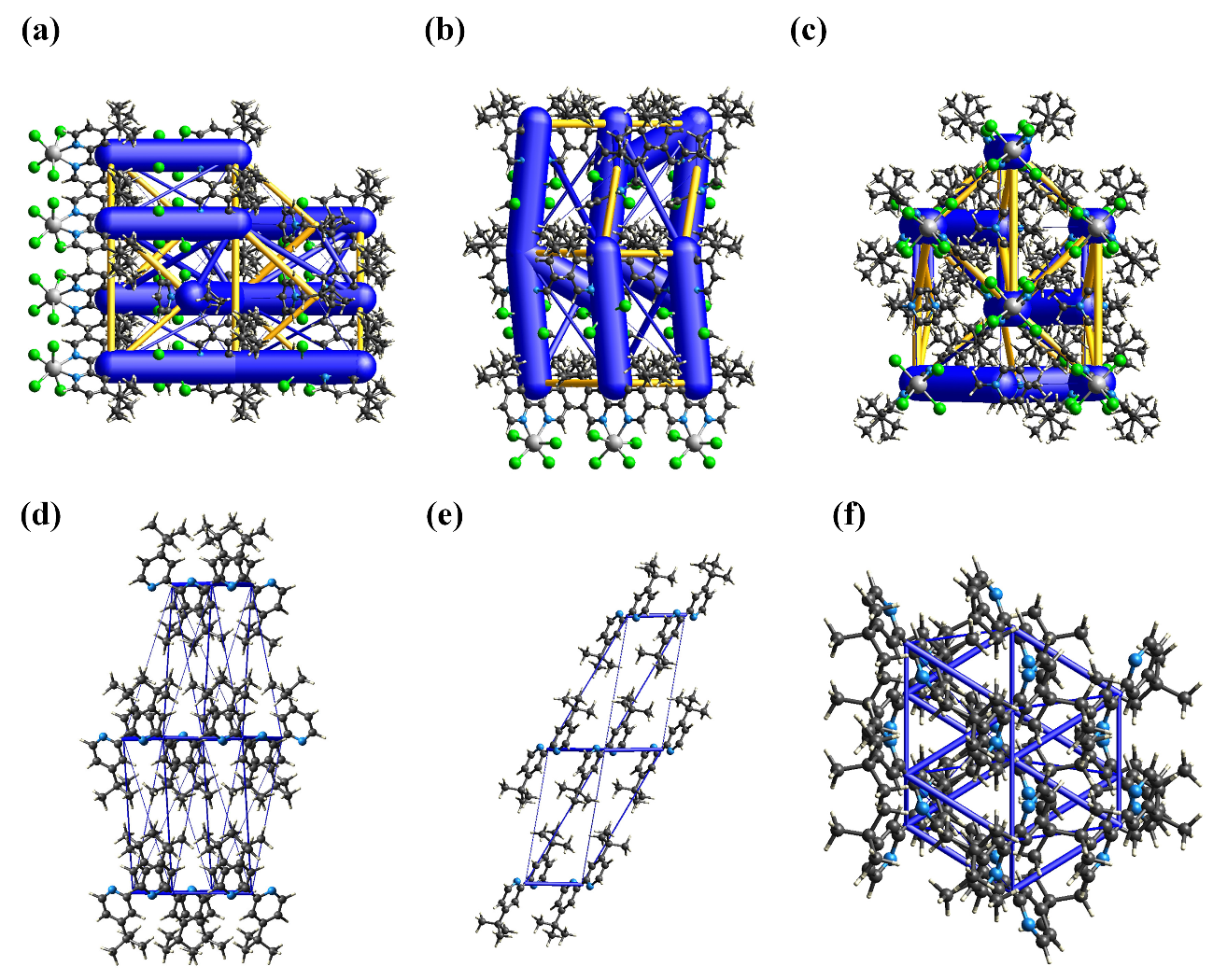


Figure S11. Simulated energy frames representing the total energy topology in [BPy][InCl_4_(dtbp)] (a-c) and B (d-f), viewed along the *a*, *b,* and *c* axis, respectively.

Table S6. The calculated interaction energy (*E*_int_).

| *E*_int_ ([BPy][InCl_4_(dtbp)]) | *E*_int_ (dtbp) |
| --- | --- |
| -409.4 kcal/mol | -137.8 kcal/mol |

Table S7. PL and CPL properties of OIMHs with non-chiral *mm*2 point groups.

| Compound | Space Group | PL Properties | CPL Properties | Ref. |
| --- | --- | --- | --- | --- |
| [BPy][InCl_4_(dtbp)] | *Pca*2_1_ | *λ*_em_ = 481 nm  PLQY = ~ 100 % | *g*_lum_ = -0.283 | This work |
| [C_8_H_12_N]_3_BiCl_6_ | *Pna*2_1_ | *λ*_em_ = 545, 548 nm | \ | ^[20]^ |
| [C_9_H_16_N_2_]PdCl_4_ | *Pmc*2_1_ | *λ*_em_ = 505 nm | \ | ^[21]^ |
| [C_9_H_16_N_2_]PdBr_4_ | *Pmc*2_1_ | *λ*_em_ = 526 nm | \ | ^[21]^ |
| HistNH_3_CoCl_4_ | *Pna*2_1_ | \ | \ | ^[22]^ |
| HistNH_3_ZnCl_4_ | *Pna*2_1_ | \ | \ | ^[22]^ |
| FAGe_0.5_Sn_0.5_Br_3_ | *Cmc*2_1_ | \ | \ | ^[23]^ |
| [(CH_3_)_4_N]_2_CuBr_4_ | *Pna*2_1_ | \ | \ | ^[24]^ |
| [Pb_2_Cl][O_2_C-(C_6_H_4_)CO_2_]_2_G | *Ima*2 | *λ*_em_ = 472 nm  PLQY = 70.4 % | \ | ^[25]^ |

C_8_H_12_N = 2-phenylethylammonium; C_9_H_16_N_2_ = 2,4,6-trimethylbenzene-1,3-diaminium; HistNH_3_ = histammonium; FA = methylammonium; (CH_3_)_4_N = tetramethylammonium, O_2_C-(C_6_H_4_)CO_2_ = hydrogen 2,6-naphthalenedicarboxylate; G = dimethylammonium

Table S8. Comparison of PLQY, *g*_lum_, and FWHM of some reported 0-D OIMHs with the present work.

| Compound | PLQY (%) | *g*_lum_ | FWHM (nm) | Ref. |
| --- | --- | --- | --- | --- |
| (*R,R*-DCDA)_3_Sb_2_Cl_12_ | 21.7 | 3.22×10^-3^ | 270 | ^[26]^ |
| (*S,S*-DCDA)_3_Sb_2_Cl1_2_ | 27.6 | -2.67×10-3 | 270 | ^[26]^ |
| *R*-(C_14_H_24_N_2_)_2_CeBr_7_ | 38.8 | 1.52×10^-2^ | 20 | ^[27]^ |
| *S*-(C_14_H_24_N_2_)_2_CeBr_7_ | 31.8 | -1.36×10^-2^ | 35 | ^[27]^ |
| *R*_2_InCl_7_ | 97.66 | 1.25×10^-3^ | 146 | ^[28]^ |
| *S*_2_InCl_7_ | 96.45 | -1.7×10^-3^ | 146 | ^[28]^ |
| (*R*)-C_6_H_15_C_l2_NO⋅SbCl_5_ | 56.3 | 2.5×10^-4^ | 120 | ^[29]^ |
| (*S*)-C_6_H_15_C_l2_NO⋅SbCl_5_ | 71.2 | -1.6×10^-4^ | 120 | ^[29]^ |
| [*R*-1,2-propanediamine(18-crown-6)_2_]SbCl_6_ | 87.2 | 5.2×10^-3^ | 116 | ^[30]^ |
| (-)-DMA_2_CsIn_0.9_Sb_0.1_Cl_6_ | 98.2 | -2.3×10^-2^ | 128 | ^[31]^ |
| *P*-DMA_4_In_0.9_Sb_0.1_Cl_7_ | 79.3 | 1.7×10^-2^ | 150 | ^[31]^ |
| [BPy][InCl_4_(dtbp)] | 100 | -0.283 | 49 | This work |

DCDA = *N,N*'-dimethylcyclohexane-1,2-diaminium; C_14_H_24_N_2_ = 1-benzyl-N,4-dimethylpiperidin-1-ium-3-aminium; *R* = *R*-2-methylpiperazine-1,4-dium; *S* = *S*-2-methylpiperazine-1,4-dium; C_6_H_15_C_l2_NO = (3-chloro-2-hydroxypropyl)trimethylammonium; DMA = dimethylammonium

### References

[1] W. M. Wendlandt, H. G. Hecht, *New York* **1966**.

[2] a) G. Kresse, J. Furthmuller, *Phys. Rev. B* **1996**, 54, 11169; b) G. Kresse, J. Hafner, *Phys. Rev. B* **1993**, 48, 13115.

[3] J. P. Perdew, K. Burke, M. Ernzerhof, *Phys. Rev. Lett.* **1996**, 77, 3865.

[4] a) M. J. Turner, S. P. Thomas, M. W. Shi, D. Jayatilaka, M. A. Spackman, *Chem. Commun.* **2015**, 51, 3735; b) M. J. Turner, S. Grabowsky, D. Jayatilaka, M. A. Spackman, *J. Phys. Chem. Lett.* **2014**, 5, 4249.

[5] L.-J. Xu, H. Lin, S. Lee, C. Zhou, M. Worku, M. Chaaban, Q. He, A. Plaviak, X. Lin, B. Chen, M.-H. Du, B. Ma, *Chem. Mater.* **2020**, 32, 4692.

[6] S. Yakunin, B. M. Benin, Y. Shynkarenko, O. Nazarenko, M. I. Bodnarchuk, D. N. Dirin, C. Hofer, S. Cattaneo, M. V. Kovalenko, *Nat. Mater.* **2019**, 18, 846.

[7] Y. Zhang, S. Yuan, Y. Yuan, Y. Bao, Q. Ran, E. Liu, J. Fan, W. Li, *Adv. Opt. Mater.* **2022**, 10, 2102041.

[8] X. Zhang, X. Jiang, K. Liu, L. Fan, J. Cao, S. He, N. Wang, J. Zhao, Z. Lin, Q. Liu, *Inorg. Chem.* **2022**, 61, 7560.

[9] Y.-Y. Ma, H.-Q. Fu, X.-L. Liu, Y.-M. Sun, Q.-Q. Zhong, W.-J. Xu, X.-W. Lei, G.-D. Liu, C.-Y. Yue, *Inorg. Chem.* **2022**, 61, 8977.

[10] Q. Wang, W. Jiang, T.-C. Liu, H.-R. Liu, R.-R. Hu, W.-X. Sun, F.-T. Guo, B. Hu, X.-W. Lei, *ACS Appl. Mater. Interfaces* **2025**, 17, 24048.

[11] J. Guan, Y. Zheng, P. Cheng, W. Han, X. Han, P. Wang, M. Xin, R. Shi, J. Xu, X.-H. Bu, *J. Am. Chem. Soc.* **2023**, 145, 26833.

[12] H.-W. Lin, A. Ablez, Z.-H. Deng, Z.-H. Chen, Y.-C. Peng, Z.-P. Wang, K.-Z. Du, X.-Y. Huang, *J. Mater. Chem. C* **2024**, 12, 5184.

[13] J. Zhou, D. Tian, W. Bai, K. Song, R. Tian, L. Aihaiti, R.-J. Xie, *ACS Appl. Mater. Interfaces* **2025**, 17, 19917.

[14] C. Sun, C.-Q. Jing, D.-Y. Li, M.-H. Dong, M.-X. An, Z.-H. Zhang, C.-Y. Yue, H. Fei, X.-W. Lei, *Adv. Sci.* **2025**, 12, 2412459.

[15] Y.-C. Peng, J.-C. Jin, Q. Gu, Y. Dong, Z.-Z. Zhang, T.-H. Zhuang, L.-K. Gong, W. Ma, Z.-P. Wang, K.-Z. Du, X.-Y. Huang, *Inorg. Chem.* **2021**, 60, 17837.

[16] Y. Deng, X. Liang, F. Li, M. Wang, Z. Zhou, J. Zhao, F. Wang, S. Liu, Q. Zhao, *Laser Photon. Rev.* **2023**, 17.

[17] K. Liu, A. Hou, J. Lin, M. Quan, Y. Xiong, Z. Guo, J. Zhao, Q. Liu, *Adv. Funct. Mater.* **2025**, 35.

[18] B. Su, G. Song, M. S. Molokeev, N. N. Golovnev, M. K. Lesnikov, Z. Lin, Z. Xia, *J. Phys. Chem. Lett.* **2021**, 12, 1918.

[19] T. R. Amarante, S. Figueiredo, A. D. Lopes, I. S. Goncalves, F. A. Almeida Paz, *Acta Crystallogr. Sect. E-Struct. Chem.* **2009**, 65, 2047.

[20] M. Essid, Z. Aloui, V. Ferretti, S. Abid, F. Lefebvre, M. Rzaigui, C. Ben Nasr, *Inorg. Chim. Acta* **2017**, 457, 122.

[21] H. Bouznif, F. Hajlaoui, K. Karoui, N. Audebrand, M. Cordier, T. Roisnel, N. Zouari, *J. Solid State Chem.* **2022**, 311.

[22] M. Wells, J. Hempel, S. Adhikari, Q. Wang, D. Allen, A. Costello, C. Bowen, S. Parkin, C. Sutton, A. J. Huckaba, *Inorg. Chem.* **2022**, 61, 17746.

[23] Y. Liu, Y.-P. Gong, S. Geng, M.-L. Feng, D. Manidaki, Z. Deng, C. C. Stoumpos, P. Canepa, Z. Xiao, W.-X. Zhang, L. Mao, *Angew. Chem., Int. Ed.* **2022**, 61.

[24] Dinesh, S. Singh, *ChemistrySelect* **2024**, 9, e202400241.

[25] C. Sun, Y. K. Li, J. L. Yin, D. Y. Li, C. Wu, C. Zhang, H. H. Fei, *Angew. Chem., Int. Ed.* **2024**, 63, e202407102.

[26] C.-Y. Chai, C.-D. Liu, B.-D. Liang, X.-B. Han, W. Zhang, C.-C. Fan, *J. Phys. Chem. Lett.* **2023**, 14, 4063.

[27] C. Li, Y. Wei, Y. Zhang, Z. Luo, Y. Liu, M. He, Z. Quan, *Angew. Chem., Int. Ed.* **2024**, 63, e202403727.

[28] L. Wang, H. Peng, Q. Wei, L. Kong, S. Yu, J. Cao, Q. Liang, J. Zhao, B. Zou, *Laser Photon. Rev.* **2025**, 19, 2400856.

[29] H.-L. Xuan, J.-L. Li, L.-J. Xu, D.-S. Zheng, Z.-N. Chen, *Adv. Opt. Mater.* **2022**, 10, 2200591.

[30] X. Han, P. Cheng, S. Han, Z. Wang, J. Guan, W. Han, R. Shi, S. Chen, Y. Zheng, J. Xu, X.-H. Bu, *Chem. Sci.* **2024**, 15, 3530.

[31] T. Song, C.-Q. Wang, H. Lu, X.-J. Mu, B.-L. Wang, J.-Z. Liu, B. Ma, J. Cao, C.-X. Sheng, G. Long, Q. Wang, H.-L. Zhang, *Angew. Chem., Int. Ed.* **2024**, 63, e202400769.
